# Fusion protein-mediated costimulation in engineered T cells: from intrinsic signaling to tumor microenvironment rewiring

**DOI:** 10.3389/fimmu.2026.1819470

**Published:** 2026-06-17

**Authors:** Ryma Toumi, Simonne J. Guenette, Shannon K. Oda

**Affiliations:** 1 Ben Towne Center for Childhood Cancer and Blood Disorders Research, Seattle Children’s Research Institute, Seattle, WA, United States; 2 M3D Graduate Program, Department of Laboratory Medicine and Pathology, University of Washington School of Medicine, Seattle, WA, United States; 3 Department of Pediatrics, University of Washington School of Medicine, Seattle, WA, United States; 4 Department of Clinical Research, Fred Hutchinson Cancer Center, Seattle, WA, United States

**Keywords:** costimulatory signaling, myeloid cell reprogramming, switch receptor, T cell engineering, tumor microenvironment, fusion protein

## Abstract

Adoptive T cell therapies have markedly improved outcomes in hematologic malignancies but their efficacy in solid tumors can be diminished by a hostile tumor microenvironment that impedes sustained therapeutic responses. Beyond challenges such as limited trafficking and antigen heterogeneity, engineered T cells face suppressive myeloid and stromal populations, inhibitory checkpoint ligand interactions, and metabolically hostile niches that collectively diminish effector function and persistence. To overcome these barriers, a new generation of fusion protein-based costimulatory strategies has emerged that couple ligand-guided sensing of the tumor microenvironment with modular control of T cell activation and fate. This review examines how conventional and non-canonical costimulatory modules, when incorporated into chimeric antigen receptor (CAR) and T cell receptor (TCR) architectures, modulate T cell differentiation and function within the tumor site. It further analyzes how membrane-anchored and secreted fusion proteins enable engineered T cells to activate dendritic cells, reprogram myeloid cells, and convert poorly inflamed tumors into treatment-responsive environments. Together, these advances establish a design framework in which fusion protein–based receptors and ligands enhance T cell function and remodel the tumor microenvironment, thereby expanding the therapeutic potential of adoptive T cell therapy for solid tumors.

## Introduction

Adoptive cell therapy (ACT), including CAR-T cells and TCR-T cells, has transformed the treatment of hematologic malignancies and is beginning to demonstrate meaningful efficacy in selected solid tumors (1, 2). Recent early-phase CAR-T studies in central nervous system (CNS) malignancies and other solid cancers have reported promising antitumor activity and manageable safety profiles (3–5). TCR-T cell therapies have likewise achieved clinically significant responses in metastatic synovial sarcoma, melanoma, and other soft tissue sarcomas, leading to an FDA approval and highlighting the broader potential of engineered cell therapies in solid tumors (6). Additionally, autologous tumor-infiltrating lymphocyte (TIL) therapy has recently been approved for advanced melanoma, with emerging evidence in lung cancer (7), demonstrating that non-engineered T cells can also be harnessed therapeutically. While many of these advances have occurred in immunologically “hot” tumors, characterized by robust T cell infiltration and an inflamed tumor microenvironment, durable responses are also emerging in traditionally “cold” or immune-excluded tumors, including certain CNS malignancies, indicating that an unfavorable baseline microenvironment can still be therapeutically remodeled (8). Nevertheless, a large fraction of solid tumors, particularly those with limited T cell infiltration and non-inflamed or highly suppressive microenvironments, continue to show inconsistent benefit. This disparity is due in part to persistent barriers imposed by the tumor microenvironment (TME), including antigen heterogeneity and a suppressive milieu with dysfunctional antigen-presenting cells (APCs), immunosuppressive myeloid cells such as tumor-associated macrophages (TAMs) and myeloid-derived suppressor cells (MDSCs), cancer-associated fibroblasts (CAFs), a dense extracellular matrix, and hypoxic niches (9, 10).

For hematologic malignancies, CD19 remains the most successful and well-characterized target for CAR-T cell therapy (11, 12). Consequently, foundational research and clinical trials have largely centered on CD19-directed therapies. Successive generations of CAR-T cells have improved activation and persistence through the incorporation of co-stimulatory domains such as CD28, 4-1BB, ICOS, or OX40, or by engineering CARs that provide antigen recognition together with costimulatory and cytokine signals intrinsically. This approach reduces dependence on antigen-presenting cells or cytokines within the TME (13). While these modifications enhance T cell proliferation, cytokine production, and resistance to exhaustion, they do not specifically address external factors in the tumor, such as suppressive immune cells and stromal barriers. This gap highlights the need for strategies that intentionally remodel the TME. Initial strategies focused on depleting suppressive immune populations, such as regulatory T cells (Tregs) and myeloid-derived cells but were constrained by toxicity, incomplete depletion, and compensatory immunosuppression (14, 15). Current efforts instead focus on engineering strategies that reshape the TME, expand the repertoire of targetable antigens, and optimize CAR-T cell design and delivery for solid tumors.

Recent reviews have summarized switch receptors (SR) and inverted cytokine receptors for T cell reprogramming in solid tumors (16, 17). Building on this foundation, we focus on fusion protein-based costimulatory strategies that rewire both intrinsic T cell signaling and the TME in engineered T cell therapies. We first summarize the functional properties of key costimulatory domains (including CD28, 4-1BB, ICOS, OX40, and others) and how their distinct signaling programs shape T cell activation, differentiation, metabolism, and persistence, as well as T cell-extrinsic outcomes such as cytokine secretion, crosstalk with antigen-presenting cells, and remodeling of the TME. We then examine platforms in which engineered T cells express membrane-bound ligands or secrete fusion proteins to activate APCs, reprogram myeloid and stromal cells, and convert “cold” tumors into inflamed, treatment-responsive tumors. We also discuss synthetic gene circuits for controlled fusion-protein delivery, safety, and translational considerations.

## Costimulatory domains in engineered T cells

**Figure 1:**
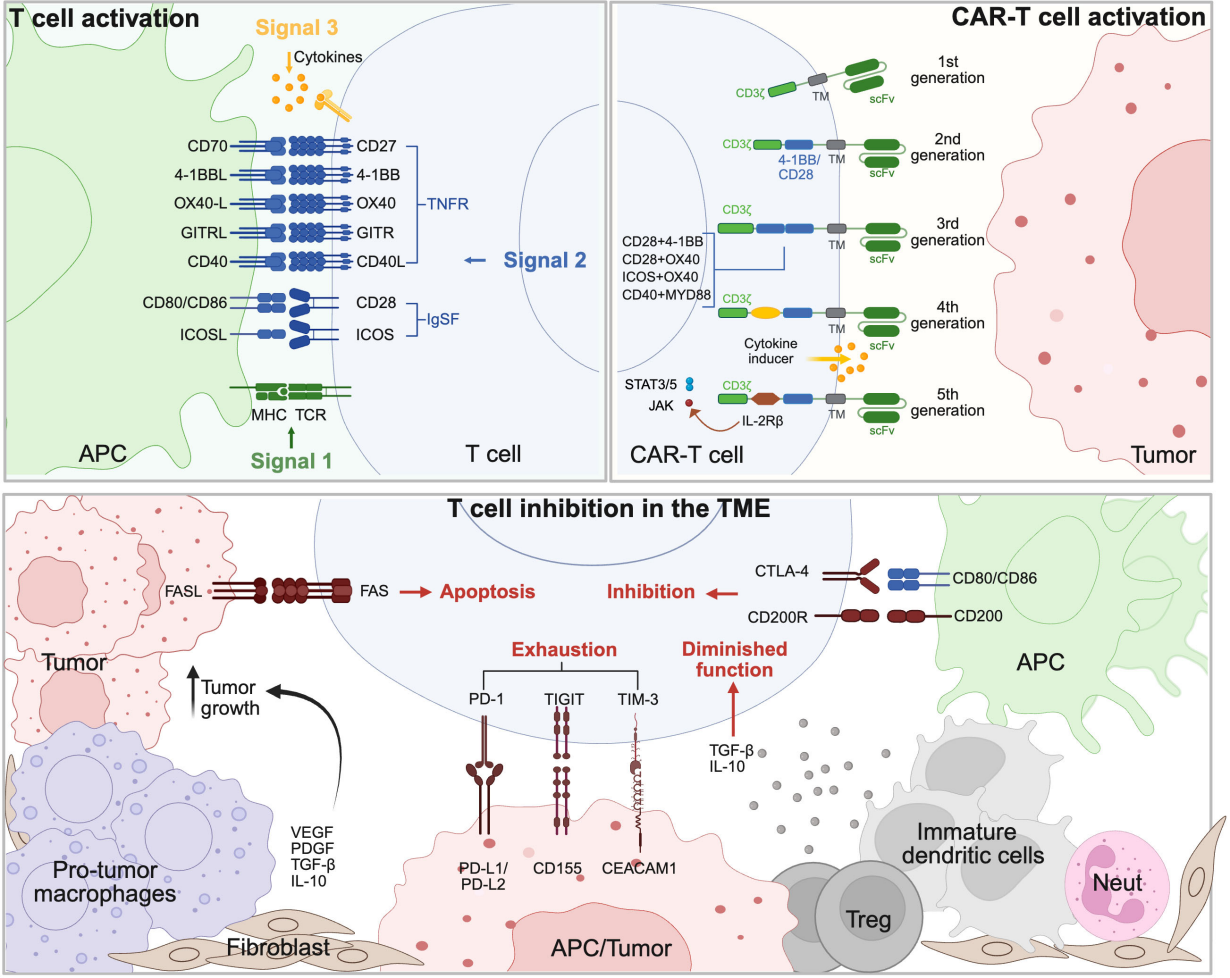
Physiologic T cell activation, CAR-T engineering, and immune suppression in the tumor microenvironment. The upper panels compare physiological T cell activation with CAR−T cell activation, highlighting signal 1 (TCR/MHC), signal 2 via costimulatory receptors, and signal 3 from cytokines. Successive CAR generations are shown to incorporate distinct costimulatory and cytokine−inducing modules. The lower panel depicts major inhibitory pathways that limit T cell function in the tumor microenvironment, including Fas-FasL-mediated apoptosis, checkpoint receptors (PD−1, TIGIT, TIM−3, CTLA−4, CD200R) that drive exhaustion or immunosuppression, and immunosuppressive cytokines and cells (pro-tumor macrophages, immature dendritic cells, neutrophils, T regulatory cells) that support tumor growth and immune evasion.

Activation of T cells is a tightly regulated process that depends on the integration of multiple distinct signals. The first signal occurs when the TCR recognizes peptide antigens presented by major histocompatibility complex (MHC) molecules on APCs (18). Although this recognition confers specificity, TCR engagement alone is insufficient for productive T cell activation and may instead result in anergy or deletion (19). The second signal, a costimulatory signal, is delivered by receptors such as CD28, a member of the immunoglobulin super-family (IgSF), which, upon binding to its ligand, CD80 or CD86, on activated APCs, amplify and sustain TCR signaling, thereby promoting T cell proliferation, effector functions, and survival (20, 21). A third signal, mediated by cytokines such as interleukin-2 (IL-2), modulates the magnitude, quality, and durability of the T cell response, influencing differentiation and fate decisions (22, 23). CD28 is the prototypic costimulatory receptor for naïve T cells and frequently provides the dominant early costimulatory signal, after which additional receptors are engaged either sequentially or in parallel. Together, these costimulatory signals fine-tune the magnitude, quality, and persistence of T cell responses, with key inputs provided by the B7-CD28 family member inducible costimulator (ICOS) and tumor necrosis factor receptor superfamily (TNFRSF) members OX40 (CD134), CD27, HVEM (CD270), GITR (CD357), and 4-1BB (CD137) (**Figure 1**) (24, 25). CD4^+^ and CD8^+^ T cells depend on distinct costimulatory pathways throughout priming, effector function, and memory maintenance. CD4^+^ T cells primarily rely on CD28-family and ICOS signaling to drive IL-2 production, differentiate into T helper (Th) subsets, and license APCs. In contrast, CD8^+^ T cells benefit more from costimulation via TNFRSF members such as 4−1BB and OX40. Although 4−1BB is expressed at similar levels on both CD4^+^ and CD8^+^ T cells, its costimulatory effects preferentially enhance the proliferation, survival, and accumulation of CD8^+^ T cells *in vitro* and *in vivo* (26). OX40 costimulation is particularly well characterized for its role in sustaining CD4^+^ effector and memory responses, reflecting higher and earlier OX40 expression on activated CD4^+^ T cells, but it can also directly enhance CD8^+^ T cell survival and memory under some conditions (27–29). Understanding these subset-specific requirements is crucial for the development of engineered cell products, as CD4- and CD8-engineered cells may require distinct costimulatory modules and activation kinetics to achieve durable, cooperative immune responses.

Traditionally, engineered T cells are equipped with either a TCR, which recognizes peptides derived from both surface and intracellular proteins when presented by MHC molecules, or a chimeric antigen receptor (CAR), which uses an antibody-derived single-chain variable fragment (scFv) to recognize accessible cell-surface antigens in an MHC-independent manner, thereby restricting CARs to a narrower set of targets than TCRs (30). In addition to conventional scFv-based CARs, recent designs employ single-domain antibody binders such as camelid variable heavy-chain domains (VHH, also known as nanobodies) as alternative antigen-recognition domains (31, 32). Nanobodies are smaller, more structurally stable and less prone to aggregation than single-chain variable fragments, helping limit antigen−independent receptor clustering while improving overall construct stability and safety. They can also be humanized to reduce immunogenicity and often recognize distinct epitopes that are inaccessible to traditional binders. Several VHH-based CARs have advanced to clinical evaluation. While many previous reviews have examined alternative CAR ectodomain architectures, this review focuses on intracellular signaling modules, with particular focus on costimulatory endodomains. First generation CAR-T cells contained only the antigen recognition domain fused to a CD3ζ activation domain and lacked costimulatory signals, resulting in limited *in vivo* expansion and persistence. Incorporation of CD28 or 4-1BB costimulatory domains in second-generation CARs markedly improved T cell proliferation, persistence, and clinical efficacy, and the downstream signaling programs of these endodomains are well documented (33, 34). Currently, seven FDA-approved CAR-T cell products employ either CD28 or 4-1BB-based costimulatory designs (35), each costimulatory domain imprinting distinct programs of expansion, persistence, and metabolism on therapeutic T cells (34, 36). The optimal selection of costimulatory endodomain depends on whether CARs are expressed in CD4^+^ or CD8^+^ T cells and on how these subsets are combined in the final therapeutic product. ICOS−based CARs have been shown to particularly enhance CD4^+^ CAR−T effector and Th1/Th17−skewed memory responses, with improved *in vivo* persistence in some models compared with CD28− or 4−1BB-based CD4^+^ CARs, while 4−1BB signaling is strongly associated with prosurvival and long−term persistence of CAR−T cells, especially within the CD8^+^ compartment (37). Notably, redirecting CD4^+^ T cells with an ICOS-based CAR can provide potent ‘helper’ support to CD8^+^ CAR-T cells, greatly enhancing their persistence and antitumor activity. This effect is especially pronounced when the ICOS intracellular domain is positioned close to the membrane, linked to the ICOS transmembrane region, or when combined with a distal 4−1BB domain in a third-generation ICOS−4−1BB design. In third-generation CAR-T cells, two costimulatory endodomains are incorporated within the cytoplasmic tail, most commonly CD28 combined with 4-1BB, or CD28 paired with other TNFRSF or ICOS-derived intracellular domains (37–39), to enable complementary signaling programs that can enhance T cell proliferation, persistence, and long-term antitumor activity (**Figure 1**).

In 2012, Hombach et al. showed that engineering CAR-T cells to deliver CD28 and OX40 signals in a configuration approximating physiological T cell activation can substantially modulate CAR-T cell function (40). In a third−generation CD28−ζ−OX40 CAR expressed in human CD4^+^ T cells, combined CD28 and OX40 costimulation selectively suppressed IL−10 production while maintaining IFNγ and IL-2 secretion, proliferation, and cytotoxicity of CAR-T cells. More recently, Guercio et al. implemented a CD28-OX40 dual-costimulatory backbone in a CD30-targeted CAR, yielding extended *in vivo* persistence and strong anti-lymphoma activity (41). In a B cell non-Hodgkin lymphoma xenograft model designed to mimic relapse by tumor re-challenge, CD28-OX40 CAR-T cells underwent robust re-expansion then contracted after lymphoma clearance, consistent with the establishment of functional memory rather than excessive, uncontrolled persistence. CD28-OX40 CAR-T platforms have entered early-phase clinical testing; third-generation GD2 directed CD28-OX40 CAR-T cells have been evaluated in early-phase trials for neuroblastoma and other GD2^+^ tumors (NCT01822652, NCT02107963, and NCT01953900). In NCT02107963, GD2-OX40-CD28/iC9 CAR-T cells were well-tolerated with no dose-limiting toxicities and a low incidence of high-grade cytokine release syndrome. However, antitumor activity was modest due to limited CAR-T expansion. Multi-omics analysis indicated that higher baseline naïve T cells and CXCR3^+^ monocytes correlated with better *in vivo* CAR-T expansion, underscoring how both product composition and the host immune milieu critically shape the effectiveness of solid−tumor CAR−T therapy (42).

More recent work has explored combining OX40 with another “late” costimulatory domain. Moreno−Cortés et al. evaluated a third-generation ROR1 CAR with tandem ICOS and OX40 domains (IOζ), which improved cytotoxicity, increased IFNγ production, and promoted a central memory (TCM) phenotype in CD4^+^ T cells compared with the 4−1BB−only CAR and the CD28/4−1BB third−generation CAR (43). In JeKo-1 xenograft models, ROR1 IOζ CAR-T cells induced rapid tumor regression, improved survival, increased TCM accumulation, and reduced exhaustion. While OX40 amplifies T cell responses, dysregulated OX40 signaling is linked to autoimmunity (44), which raises general safety considerations when incorporating this domain into CAR constructs, especially regarding on-target, off-tumor effects. However, in the reported preclinical models no autoimmune−like toxicity has been specifically attributed to the ROR1−IOζ construct, and clinical data will be required to define the true risk profile.

In addition to traditional costimulatory domains, other groups have equipped CARs with unconventional signaling modules to improve T cell persistence, proliferation, memory and resistance to exhaustion. One unconventional example is the integration of B cell-associated signaling motifs. Julamanee et al. developed a CD19 CAR featuring a chimeric CD79A/CD40 costimulatory domain (CD19.79a.40ζ), benchmarked against standard CD28- and 4-1BB-based designs (45). In B cells, CD79A, a B cell receptor (BCR) coreceptor, and CD40 synergistically activate signaling pathways, including NF-κB, NFAT, and AP-1. When introduced into CAR-T cells, this composite endodomain led to amplified and sustained NF-κB/p38 and NFAT signaling upon antigen exposure. Functionally, CD19.79a.40ζ CAR-T cells demonstrated vigorous proliferation independent of exogenous IL-2, maintained cytotoxic activity at low effector-to-target ratios *in vitro*, and exhibited superior tumor control and overall survival in NALM-6 and Raji xenograft models compared to their CD28ζ and 4-1BBζ counterparts. In a complementary strategy, MyD88/CD40 CARs have also demonstrated enhanced proliferation following antigen engagement, preservation of a less differentiated, memory−like phenotype, and improved effector functions *in vitro* and *in vivo* compared with CD28− or 4−1BB−based CARs (46). Collectively, these studies suggest that the integration of B cell-derived signaling, which synergizes with T cell pathways, particularly via the incorporation of B cell signaling modules such as CD79A/CD40 or MyD88/CD40, can improve CAR-T cell fitness, promote sustained proliferative capacity, and enhance antitumor efficacy (45–48). Notably, prostate stem cell antigen (PSCA)−directed CAR−T cells co−expressing an inducible MyD88/CD40 (iMC) costimulatory switch have progressed to early−phase clinical testing (NCT02744287), in which pharmacologic activation of the iMC module with rimiducid augmented CAR−T expansion and function in patients with solid tumors (48), showing the clinical applicability of drug−tunable costimulation. The trial was ultimately suspended because the level of MyD88/CD40 activation required for efficacy was associated with safety concerns and an unfavorable overall risk–benefit profile (49). This GoCAR−T platform represents the first clinical application of a MyD88−based costimulatory switch in CAR−T cells and thus the observed toxicities may be attributable to supra−physiologic MyD88 activation.

As mentioned earlier, effective T cell activation requires three signals, the third being cytokines. However, systemic cytokine delivery is challenging because these small, rapidly cleared proteins require pharmacokinetic optimization to prolong their half−life and maintain therapeutically relevant concentrations in the tumor microenvironment while minimizing off−tumor toxicity. In this context, fourth−generation CAR−T cells, also known as ‘TRUCKs’ (T cells redirected for universal cytokine−mediated killing), are engineered to secrete cytokines such as IL−12 or IL−18 and can be further enhanced with chemokine receptors or other modulatory elements to promote better tumor infiltration, overcome immunosuppression, and stimulate endogenous immune responses (50). Fifth−generation CAR−T cells build on this concept by incorporating, into a second−generation CAR backbone, an additional cytokine receptor–derived domain that couples antigen recognition to JAK/STAT signaling, thereby bypassing the need for autocrine cytokine secretion and more directly programming T cell fate (**Figure 1**). Kagoya et al. developed a next-generation CD19 CAR that couples CD3ζ/CD28 signaling with a truncated IL-2 receptor β chain containing a STAT3-binding YXXQ motif. This design enables antigen-dependent JAK-STAT activation and promotes JAK and STAT3/STAT5 signaling, thereby enhancing proliferation while restricting terminal differentiation *in vitro* (51).

Unlike CAR-T cells, which target only cell surface antigens, TCR-T cell therapy can recognize intracellular or membrane-associated proteins processed and presented by MHC molecules, thereby expanding the range of targetable tumor antigens. This capability enables targeting of aberrantly expressed intracellular targets, including transcription factors such as Wilms’ Tumor Antigen 1 (WT1) (52), overexpressed tumor-associated antigens, driver mutations, splice-derived neoantigens, cancer-testis antigens such as New York esophageal squamous cell carcinoma 1 (NY−ESO−1), Melanoma Antigen Genes (MAGEs) (53), as well as minor histocompatibility antigens such as HA−1 (54). Additionally, TCR-T cells also respond to lower densities of peptide-MHC complexes, offering potential benefits in tumors with low antigen expression (55, 56). In contrast to second- and later generations CARs, which have built-in co-stimulatory domains, TCRs rely on separate co-stimulatory receptors, making them promising candidates for synthetic engineering of integrated co-stimulatory modules. A recent phase I clinical study evaluated MAGE-A1-specific TCR-engineered T cells in patients with solid tumors (NCT04639245) (57). To address the limited efficacy of first-generation TCR-T therapy, the study incorporated a CD8/CD28 chimeric co-receptor by fusing the CD28 intracellular domain to the CD8β chain, which enhanced cytokine production, persistence, and intratumoral accumulation of both CD4^+^ and CD8^+^ T cells in preclinical models while reducing exhaustion and improving tumor control (57). This approach was further refined by introducing a mutated CD28 cytoplasmic domain (TCR-MA1-CD8/CD28mut) that targets YMNM, PRRP, and PYAP motifs, previously implicated in CAR-T cell signaling (58). The engineered variant (TCR-MA1-CD8/CD28_mut_) demonstrated increased resistance to exhaustion after repeated antigen-specific stimulation, suggesting that fine-tuning CD28 signaling can optimize the balance between robust T cell activation and maintenance of stem-like properties in TCR-based therapies (57).

Collectively, these strategies demonstrate that integrating conventional and non-canonical co-stimulatory domains and cytokine circuits can improve T cell fitness and partially mitigate suppression in the hostile TME. Yet, in solid tumors, suppressive pathways are influenced by inhibitory immune checkpoint receptors that dampen TCR and CD28 signaling, even with optimized CAR or TCR designs. This challenge has driven engineering efforts to reprogram checkpoint signals, converting PD-1, CTLA-4, TIM-3, TIGIT, CD200R, Fas, and similar receptors from inhibitory pathways into intrinsic co−stimulatory inputs (**Figure 1**). Oda et al. and others have developed switch receptors that fuse inhibitory extracellular domains to various costimulatory endodomains, thereby rerouting engagement of these checkpoints from suppressive signaling to T cell activation (59–61). Oda et al. further refined these constructs by optimizing features such as dimerization motifs and ectodomain length so that switch receptors localize efficiently to the immune synapse, providing a robust platform for subsequent fusion−protein engineering in T cells (60, 61). These advances rely on fusion receptors assembled from native or minimally modified protein domains, ensuring signaling is limited to engineered T cells at ligand engagement sites and lowering immunogenicity compared to fully synthetic constructs. This design is especially important for allogeneic applications, where constructing switch receptors from native human domains may help reduce host immune recognition of the engineered receptor. Additionally, SRs could improve short-lived allogeneic T cell therapies effectiveness by providing immediate, ligand-dependent costimulation that enhances effector functions during the limited period of persistence in the host. In the next sections, we discuss checkpoint switch receptors as a versatile platform for intrinsic costimulation and describe fusion protein−based strategies that apply similar principles to reprogram the TME.

## Reprogramming checkpoint inhibitory pathways using switch receptors to enhance T cell activation and tumor control

### PD-1-based switch receptors

Programmed cell death protein−1 (PD−1; CD279) is an inhibitory checkpoint receptor. It is rapidly upregulated on activated T cells, where it limits CD28-mediated co-stimulation (62) and dampens TCR−driven effector function (63). In the TME, tumor and stromal cells often express PD−L1 (B7-H1; CD274) and PD−L2 (B7-DC; CD273), either constitutively or in response to inflammatory cytokines like IFNγ and TNF-α (64). When PD-1 is engaged, it recruits SHP−1 and SHP-2 phosphatases, which dephosphorylate proximal TCR components, the CD28 costimulatory receptor, and the common cytokine receptor γ chain (γc) (65), thereby suppressing TCR and costimulatory signaling, reducing proliferation and cytokine production, and ultimately driving T cell exhaustion (66, 67).

Therapeutic targeting of PD-1 began with monoclonal antibodies that block PD-1/PD-L1 interactions. Immune checkpoint blockade (ICB) has significantly improved outcomes in several cancer types (68), and multiple ICBs are now FDA-approved for indications like melanoma and non-small-cell lung cancer (NSCLC) (69–71). However, most patients do not experience durable responses, and even tumors with high PD-L1 levels, such as certain NSCLC subsets, may not respond. Notably, PD-L1 expression on tumor-infiltrating immune cells, especially TAMs, correlates more strongly with clinical benefit than expression on tumor cells alone, highlighting myeloid PD-L1 as a key determinant of efficacy (68, 72, 73).

**Figure 2:**
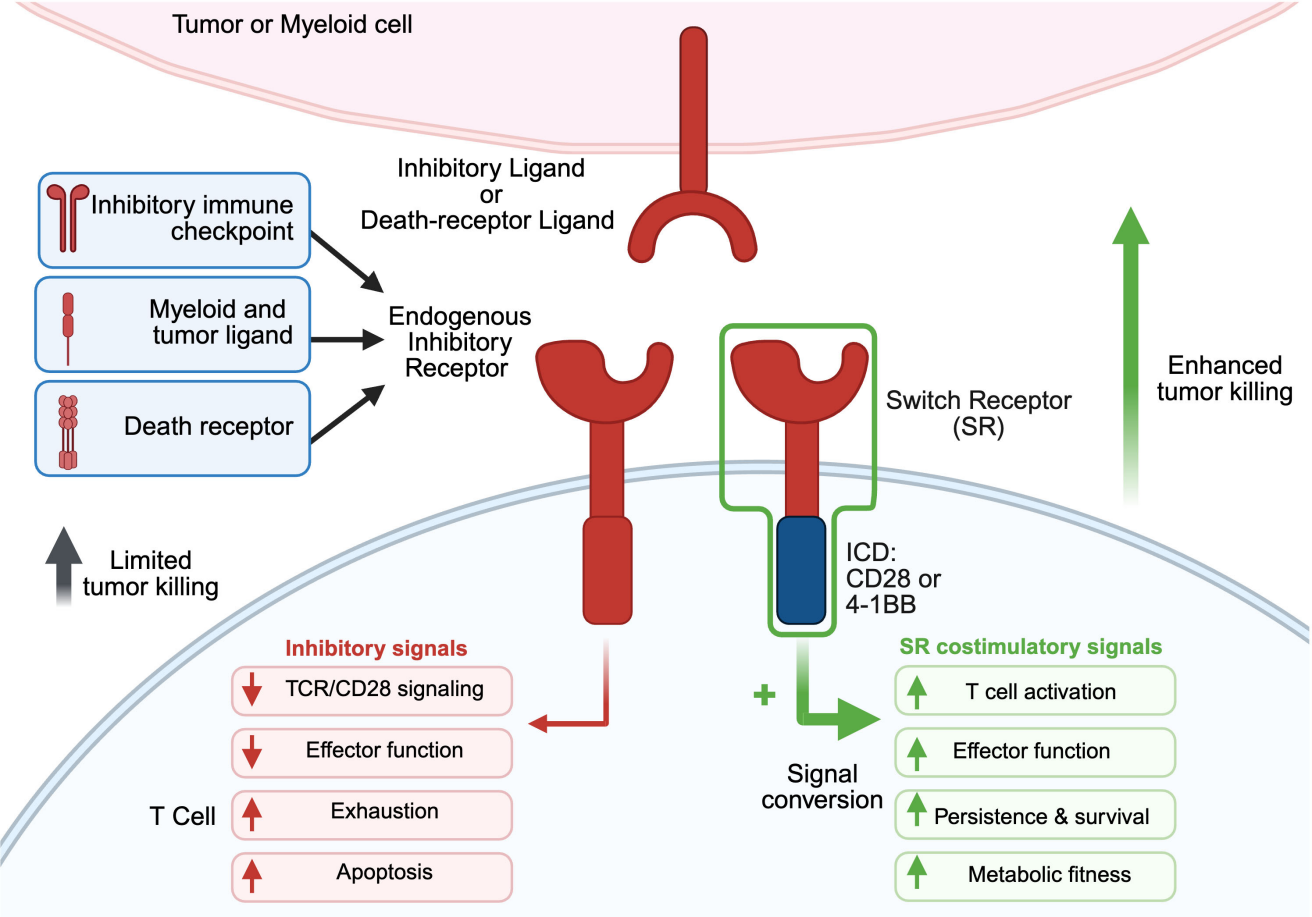
Schematic overview of switch receptors (SR) strategies that reprogram inhibitory or pro-apoptotic signals into costimulatory signals in engineered T cells. Tumor or myeloid cells within the tumor microenvironment express inhibitory immune checkpoints, myeloid- and tumor-derived ligands, or death receptor ligands that engage endogenous inhibitory receptors on T cells, resulting in reduced TCR/CD28 signaling, diminished effector function, increased exhaustion, and apoptosis, collectively leading to limited tumor killing. In contrast, engineered switch receptors (SRs) preserve the extracellular domain specificity for these inhibitory or death ligands but replace the endogenous intracellular domain with a costimulatory ICD (for example, CD28 or 4−1BB), thereby converting ligand engagement into a positive signal. ICD: intracellular costimulatory domain.

PD-1-directed therapy is intended to restore effector function in exhausted T cells (74), but these antibodies also reshape the TME, particularly the myeloid compartment. Macrophages, which are efficiently recruited to and enriched within tumor tissue, are broadly categorized along an M1–M2 spectrum: M1-like macrophages are pro-inflammatory and support anti-tumor immunity, while M2-like macrophages are anti-inflammatory and promote tumor growth (75). Though this classification is recognized as overly simplistic given the phenotypic and functional diversity of TAMs (76, 77), it helps explain how macrophage states influence ICB outcomes. In many solid tumors, PD-L1 is upregulated on M2-skewed TAMs as disease progresses; these cells deliver strong PD-1-mediated inhibitory signals to CD8^+^ T cells and secrete immunosuppressive cytokines such as IL-10, TGF-β, and other mediators, reinforcing T cell exhaustion and exclusion from the tumor bed. Clinical and translational evidence shows that macrophage expression of PD-L1 can predict response to PD-1/PD-L1 blockade as well as, or better than, tumor cell PD-L1 (78). PD-1/PD-L1 antibodies can reprogram PD-L1^+^ TAMs toward a more inflammatory, M1-like phenotype, enhancing antigen presentation and cytokine production. Conversely, M2−polarized tumor−associated macrophages can promote resistance to PD−1 blockade by using Fcγ receptors to capture therapeutic anti−PD−1 antibodies from PD−1^+^ T cells, thereby reducing PD−1 occupancy by the drug and allowing inhibitory PD−1/PD−L1 signaling to resume (79, 80). Thus, a substantial portion of the efficacy and toxicity of these agents arises from their impact on myeloid and tissue−resident cell compartments, not just within T cell populations. As a result, antibody−based checkpoint blockade has limitations in tumors with high immunosuppressive myeloid cells, underscoring the need for alternative strategies. One such approach is cell−intrinsic engineering to reprogram checkpoint pathways. Switch receptors (SRs) exemplify this strategy by converting engagement with inhibitory ligands, such as PD−L1, into signals that enhance T cell activation within TME (**Figure 2**).

The first PD-1-CD28 switch receptor, developed by Prosser et al., retained effective PD-L1 binding while delivering potent costimulatory signals, resulting in increased cytokine secretion, proliferation, and effector function (59). Subsequent studies showed that PD-1-CD28 receptors enhanced CAR-T cell control of solid tumors (81, 82) and improved cytokine production and cytotoxicity in low-avidity TCR-engineered T cells across a range of PD-L1-positive targets (83). This versatility highlights the utility of PD-1-CD28 switch receptors for both CAR- and TCR-based therapies. Early clinical data further support the promise of this approach (**Table 1**). In a Phase I trial of 14 patients with recurrent PD−L1^+^glioblastoma refractory to surgery and radiotherapy (NCT02937844), PD−1-CD28−engineered T cells increased intratumoral T cell infiltration and IFN−γ/IL−6 levels in cerebrospinal fluid, all while maintaining a manageable safety profile (84). Additional studies (NCT02930967, NCT03258047) have also reported encouraging antitumor activity of CD19 CAR-T cells co-expressing PD-1-CD28 in both solid tumors and B cell lymphomas, though larger trials are needed to define durability, safety, and optimal patient selection (85–87). To further broaden costimulatory signaling and enhance antitumor responses, PD-1 switch receptors have been engineered to signal through alternative intracellular domains, such as 4-1BB, generating PD-1-4-1BB switch receptors. Preclinical studies show that these receptors enhance the function of PRAME-specific TCR-T cells in melanoma (88), and when co-expressed with second-generation HER2 CAR-T cells, improve activation and clearance of pleural and peritoneal metastases in xenograft mice - findings which have motivated initiation of a Phase I clinical trial (NCT04684459) (89). More recently, PD−1-based switch receptors have been expanded to engage alternative costimulatory pathways, such as the CD2-CD58 axis. Loss of CD2 on CAR-T cells or its ligand CD58 (LFA-3) on tumors diminishes antigen avidity and costimulation; restoring CD2 signaling with a PD1-CD2 switch receptor rescues effector function and improves tumor control in preclinical models (90).

**Table 1:** Clinical trials of switch receptors in adoptive T cell therapy.

Switch receptor	Engineered T cell platform	Clinical identifier	Clinical phase	Patient population
PD-1-CD28_tm+ICD_	Anti-PD-L1 CAR-T cells	NCT02937844	I	Recurrent GBM
PD-1-CD28_tm+ICD_	Anti-CD19 CAR-T cells	NCT02930967	I	Recurrent or Metastatic Malignant Tumors
PD-1-CD28_tm+ICD_	Anti-CD19 CAR-T cells	NCT03258047	I/II	Relapsed/​Refractory B Cell Lymphoma
Fas-4-1BB	(HLA-A)*11:01-restricted Kirsten rat sarcoma (KRAS) G12V-specific transgenic TCR-T cells	NCT06105021	I	Advanced or Metastatic Solid Tumor

### CTLA-4-based switch receptors

CTLA-4 (CD152) is a type I transmembrane inhibitory receptor in the immunoglobulin superfamily (91). It is induced on activated CD4^+^ and CD8^+^ T cells and constitutively expressed at high levels on regulatory T cells, where it is critical for their suppressive function. Structurally similar to the costimulatory receptor CD28, CTLA-4 binds the same ligands, CD80 (B7-1) and CD86 (B7-2), with significantly higher affinity and avidity, allowing CTLA-4 to outcompete CD28 for B7 engagement, thereby antagonizing costimulation (92). Upon ligand binding, the cytoplasmic tail of CTLA-4 becomes phosphorylated and recruits phosphatases such as SHP-2 and PP2A. These phosphatases dephosphorylate proximal TCR signaling components, including CD3ζ and LAT, and attenuate PI3K-Akt and Ras-MAPK pathways, resulting in reduced activation of NFAT, NFκB, and AP-1 as well as diminished cell cycle progression and cytokine production. Beyond these cell-intrinsic effects, CTLA-4 also exerts extrinsic suppression by downregulating CD80/CD86 on APC through transendocytosis and by inducing immunoregulatory programs in DCs. Collectively, these mechanisms limit CD28-dependent costimulation and restrain effector T cell responses (93).

The development of a CTLA-4-CD28 switch receptor was motivated by concerns that simply overexpressing a decoy CTLA-4 ectodomain on T cells could compete with CD28 for CD80/CD86 binding, and thereby inhibit, rather than enhance, T cell activation. Previous studies demonstrated that substituting the SHP2-binding motif of CTLA-4 (GVYVKM) with the analogous CD28 motif (SDYMNM) converted CTLA-4 from an inhibitory receptor to one that promoted IL-2 production in T cell hybridomas (94). Shin et al. fused the CTLA-4 ectodomain to the CD28 intracellular domain generating a CTLA-4-CD28 switch receptor (95). In murine T cells, this receptor increased cytokine production *in vitro* and enhanced tumor regression after adoptive transfer in two syngeneic tumor models. The most pronounced benefit was observed when both CD4^+^ and CD8^+^ T cells were engineered, with CD4^+^ cells exhibiting increased IL-2 secretion, which is essential for antitumor activity. A similar CTLA−4-CD28 switch receptor design was subsequently applied in the allogeneic setting by Park et al., who used donor−derived T cells expressing CTLA−4/CD28 switch receptor to enhance graft−versus−tumor activity in models of relapsed hematologic malignancies (96). Later, Lin et al. developed a CTLA−4-CD28-CD3ζ receptor, termed CTLA-4 CAR, which couples CTLA−4 ligand engagement to both CD28 costimulation and CD3ζ signaling, specifically targeting CD80/CD86^+^ B−cell malignancies (97). CTLA-4 CAR-T cells secreted higher levels of IL−2 and IFNγ, exhibited enhanced cytotoxicity against CD80/CD86^+^ leukemia and lymphoma cells, and reduced tumor burden, in both xenograft and syngeneic models. Furthermore, the CTLA-4 CAR mitigated local immunosuppression in the TME by depleting CD80/CD86−positive myeloid−derived suppressor cells. Nevertheless, the potential of this design is limited by findings that CTLA-4 CAR-T cells induced mild, non−lethal graft−versus−host-like symptoms and cytokine release syndrome in mice. These observations suggest that CTLA-4 CAR-T cells may still pose safety concerns for clinical application, even in autologous settings and highlight the need for further preclinical and clinical evaluation. In patients with diffuse large B cell lymphoma (DLBCL) treated with CD19 CAR-T cell therapy, CD80 and CD86 are frequently upregulated on both lymphoma cells and lymph node B cells, suggesting that many tumors express abundant CTLA-4 ligands following adoptive cell transfer. This raises the possibility that CTLA-4-based switch receptors may interact with ligands on both malignant and normal B cells, potentially increasing the risk of on-target, off-tumor toxicity. To improve selectivity, Prinz et al. combined a first-generation CD19 CAR lacking intrinsic costimulation with a CTLA-4-4-1BB switch receptor, creating an AND-gate mechanism that requires simultaneous recognition of CD19 and CD80/CD86 for full activation (98). In preclinical models, this CAR/SR strategy spared CD19^+^CD80^-^CD86^-^ healthy cells and reduced B cell aplasia and immune effector cell-neurotoxicity syndrome, while maintaining potent antitumor activity.

### TIM-3-based switch receptors

TIM−3 (T cell immunoglobulin and mucin domain−containing protein 3) is an inhibitory checkpoint receptor upregulated on activated T cells, NK cells, myeloid cells, and Tregs, where it negatively regulates Th1 and CD8^+^ T cell activity. Although the TIM−3 cytoplasmic tail lacks classical inhibitory motifs, binding of ligands such as galectin-9, HMGB1, phosphatidylserine, and CEACAM1 leads to phosphorylation of conserved tyrosines and triggers the release of BAT3 and recruitment of negative regulators, thereby suppressing T cell activation or inducing Th1 cell death (99, 100).

TIM−3 is highly expressed on tumor-infiltrating T cells in many solid tumors (101), and several TIM−3-targeting antibodies are in clinical development (102). However, few TIM-3-targeted switch receptors have been engineered, indicating a largely unexplored potential for CAR- and TCR-T therapies. The first TIM-3-CD28 switch receptor, developed by Zhao et al. (103), demonstrated improved cytokine secretion, cytotoxicity, and persistence of anti-CD19 CAR-T cells in mice, with reduced exhaustion and no overt toxicity. Subsequent single-cell profiling confirmed improved cytokine production and reduced exhaustion compared to conventional CAR-T cells (104). Building on these findings and the CD200R-switch receptor engineering strategy (discussed in detail below) developed by Oda et al., Blaeschke et al. further optimized the TIM−3-CD28 switch receptor, illustrating the broader applicability of these design principles to SR development (105). Notably, TIM−3-CD28 SRs increased the proliferative capacity of both first- and second-generation CD19 CAR-T cells, with enhanced responses observed even under weak or polyclonal stimulation, raising the possibility of tonic or ligand−independent signaling. However, this proliferation was not consistently matched by increased cytokine secretion, especially in second-generation CAR-T cells, highlighting a potential dissociation between expansion and effector function. These findings underscore the need for careful safety assessment and validation of TIM-3-CD28 switch receptors across additional CAR targets and disease models.

### TIGIT-based switch receptors

TIGIT (T cell immunoglobulin and ITIM domain) is an inhibitory immune checkpoint receptor highly expressed on activated and exhausted T cells and NK cells in the TME. By binding its primary ligand CD155 (PVR) and other nectin family members (CD112, CD113, nectin-4), TIGIT suppresses antitumor immunity (106). CD155 is frequently overexpressed on both tumors and myeloid cells, further promoting TIGIT-mediated suppression. When engaged, TIGIT transmits inhibitory signals that affect NK and T cells differently. In NK cells, TIGIT phosphorylation recruits phosphatases via its cytoplasmic immunoreceptor tyrosine-based inhibitory motif (ITIM), directly suppressing cytotoxicity through an ITIM-dependent manner (107). In T cells, a purely ITIM−mediated inhibitory pathway has not been clearly demonstrated, and the mechanisms by which TIGIT attenuates TCR signaling remain incompletely defined. Proposed mechanisms include competition with the costimulatory receptor CD226 (also known as DNAM-1) for CD155 and cell-intrinsic negative signaling that limits proliferation and cytokine production (108). Additionally, TIGIT-CD155 interactions on APCs induce an anti-inflammatory, tolerogenic phenotype, which further dampens T and NK cell cytotoxicity in the TME (106).

In preclinical models, CAR− or TCR−engineered T cells co-expressing an optimized TIGIT-CD28 switch receptor, in which the TIGIT ectodomain and transmembrane region are fused to the CD28 intracellular signaling domain, exhibited enhanced effector function against CD155−positive tumors. This finding shows that inhibitory TIGIT-CD155 interactions can be rewired into potent costimulatory signals to boost T cell-based immunotherapies (109). Co-expression of PD-1 and TIGIT on tumor-infiltrating T cells is associated with poor prognosis and accelerated tumor progression. Comparative analyses of healthy, inflamed, and malignant tissues have revealed high co-expression of these inhibitory receptors, supporting the rationale for dual checkpoint inhibition (110). Notably, combining PD-L1 and TIGIT blockade has yielded greater clinical benefits than PD-L1 inhibition alone in patients with PD-L1-positive non-small cell lung cancer, without increasing toxicity. However, several late-stage trials of anti-TIGIT antibodies combined with PD-1/PD-L1 blockade have been halted or failed to meet primary endpoints (111). Engineered SRs offer an alternative strategy by directly converting inhibitory signals into T cell-intrinsic activation rather than relying solely on extracellular antibody blockade. To exploit the potential synergy between TIGIT/CD155 and PD-1/PD-L1 axes, Zhao et al. engineered a dual PD−1/TIGIT/CD28 switch receptor by fusing the extracellular portions of PD−1 and TIGIT to the CD28 transmembrane and signaling domains, and co-expressed this receptor with a 4−1BB−based anti−EGFR CAR (112). This design augmented cytokine secretion, proliferation, and cytotoxicity *in vitro*. In both xenograft and patient−derived mouse models, SR−expressing CAR-T cells slowed the tumor growth, reduced tumor burden, prolonged survival, and resisted tumor rechallenge, illustrating the therapeutic promise of TIGIT−based SRs.

## Switch receptors targeting death and myeloid checkpoints

### CD200R-based switch receptors

CD200R is an inhibitory Ig−superfamily receptor primarily found on myeloid cells, including macrophages, DCs and myeloid−derived suppressor cells, as well as on activated T cells (113, 114). Its ligand, CD200, is a type I transmembrane glycoprotein widely expressed on lymphocytes, neural and endothelial cells. Notably, CD200 is upregulated in several malignancies such as myeloid leukemia (AML) (115), neuroblastoma (116), melanoma (117), and a broad range of neuroendocrine tumors, including small cell lung carcinoma (118). Although CD200R lacks classical ITIM motifs (119), its cytoplasmic tail contains three tyrosines, one within an NPXY motif that is phosphorylated upon CD200 binding, leading to recruitment of Dok−1 and Dok−2 and association with RasGAP and SHIP; this cascade dampens ERK, p38 and JNK activation and suppresses myeloid cell activity (120, 121). In T cells, engagement of CD200R by CD200 shifts cytokine production from a Th1− to a Th2-polarized profile, promotes induction of Tregs and suppresses cytotoxic T cell responses, collectively limiting tumor−specific effector immunity (122, 123).

Oda et al. engineered a panel of CD200R−based immunomodulatory fusion proteins (CD200R-CD28 IFPs) in which the CD200R ectodomain is fused to a CD28 costimulatory signaling domain, with the goal of overcoming inhibitory CD200R signaling in CD200^+^ leukemia (60). This work demonstrated that CD200, which is frequently upregulated on AML blasts and leukemia stem cells, suppresses human T cell responses, can be repurposed as a source of CD28 costimulation when recognized by a CD200R-CD28 IFP on tumor−specific T cells. Structure–function studies evaluated CD200R IFP variants differing in ectodomain length, CD28 dimerization motifs, and a CD28 glycosylation site. Constructs that retained an ectodomain size compatible with entry into immunological synapse and preserved the dimerizing CD28 cysteine provided the strongest enhancement of T cell activation. Collectively, these findings indicate that CD200R-CD28 switch receptors can convert an inhibitory checkpoint signal into productive costimulation and offer general design principles for developing CD28−based fusion receptors targeting other inhibitory ligands.

### Fas-based switch receptors

Fas (CD95, APO−1, TNFRSF6) is a type I transmembrane receptor from the TNF receptor superfamily that is broadly expressed across cell types, with particularly high levels on activated T cells and other immune cells such as macrophages. Fas contains a cytoplasmic death domain (DD), which initiates apoptosis when engaged by its ligand (124). The ligand, Fas ligand (FasL, CD95L), is a type II transmembrane protein that trimerizes and binds to Fas, triggering the recruitment of FADD and procaspase−8 to form the death−inducing signaling complex (DISC), ultimately activating caspase−3 and inducing cell death (125). Membrane-bound FasL can be cleaved by metalloproteases to produce soluble trimers; this soluble form lacks full apoptotic activity and can even antagonize the function of membrane-bound FasL (126, 127). Fas/FasL interactions are essential for immune homeostasis (128): they mediate deletion of activated peripheral T cells, enforce activation-induced cell death (AICD) after repeated TCR stimulation (129, 130), and contribute to immune privilege in sites such as the eye and testis (131).

Within tumors, Fas-FasL signaling is highly context-dependent and can produce seemingly opposite outcomes. In the “Fas counterattack” model, FasL expressed by malignant or stromal cells induces apoptosis in Fas−positive tumor−infiltrating lymphocytes, generating an immune−privileged niche that facilitates immune escape. FasL expression has been reported in many cancers, including breast (132), ovarian (133), liver (134), and melanoma (135), often correlating with poor prognosis and reduced T cell infiltration (136). However, the true prevalence of tumor−cell FasL expression remains debated, as some studies have failed to detect FasL and several widely used anti−FasL antibodies have shown limited specificity. Moreover, enforced FasL expression in tumors does not invariably promote immune evasion; in some models, it instead triggers a robust neutrophil−mediated inflammatory response that results in tumor rejection (137, 138). Conversely, sustained loss of Fas or FasL in certain models induces “death induced by CD95R/L elimination” (DICE)−mediated tumor cell death, revealing a survival dependency on Fas signaling in some contexts (139, 140). Taken together, Fas/FasL can drive either immune suppression or tumor cell death depending on tumor type, microenvironment, and pathway engagement, complicating efforts to therapeutically target this axis.

In adoptive T cell therapy, transferred human T cells are often Fas-positive and thus vulnerable to FasL-mediated apoptosis within the TME (141). To overcome this limitation, Yamamoto et al. (142) engineered dominant-negative Fas receptors (Fas DNRs) that lack apoptotic signaling capacity. Both mouse and human T cells expressing Fas DNRs demonstrated markedly improved persistence in peripheral tissues and tumors. This enhanced persistence translated into superior tumor regression and overall survival in both solid and hematologic syngeneic cancer models, including B16 melanoma and B cell acute lymphoblastic leukemia. Importantly these benefits were achieved without evidence of uncontrolled lymphoproliferation or autoimmunity, even several months after transfer.

To harness the abundant FasL in the TME while protecting therapeutic T cells from Fas−mediated apoptosis, Oda et al. engineered a Fas-4-1BB switch receptor (61). This construct fuses a decoy Fas receptor with the 4-1BB intracellular domain, converting a death signal into a pro-survival cue (**Figure 2**). In murine T cells, Fas-4-1BB enhanced prosurvival signaling, proliferation, cytotoxicity, and mitochondrial fitness, including increased mitochondrial biogenesis and oxidative metabolism. These improvements supported superior *in vitro* function and improved *in vivo* persistence, leading to enhanced therapeutic efficacy in a murine model of AML (FBL). To test this approach in a stringent solid−tumor setting, Oda et al. used the KrasLSL-G12D/+;Trp53LSL-R172H/+;p48Cre/+ (KPC) model of pancreatic ductal adenocarcinoma (PDAC) (61). In this model, mice spontaneously develop autochthonous pancreatic tumors within a highly immunosuppressive, FasL−rich, mesothelin (MSLN)−high microenvironment that closely mimics human PDAC. Earlier studies showed that TCR-MSLN-tumor-infiltrating T cells initially mount an antitumor response but progressively become dysfunctional (143). Expression of Fas−4−1BB in TCR-MSLN-T cells significantly improved persistence and survival, resulting in superior tumor control and prolonged overall survival compared to TCR−only ACT (61). Building on these findings, Anderson et al. (144) evaluated Fas−4−1BB in mesothelin−specific TCR−engineered T cells for ovarian cancer. This study also showed enhanced intratumoral accumulation, cytokine production, and tumor clearance in mice, as well as improved function of human MSLN−TCR-T cells *in vitro*. Collectively, these preclinical advances paved the way for the first-in-human Phase I trial (NCT06105021) of AFNT−211, an autologous KRAS G12V−specific TCR−T product coexpressing CD8α/β and a FAS−4−1BB switch receptor, in adult patients with solid tumors harboring a KRAS G12V mutation. This mutation is often found in non-small cell lung cancer (NSCLC), colorectal cancer (CRC), and PDAC (**Table 1**). Beyond 4-1BB, the Fas ectodomain has been combined with other TNFR superfamily co-stimulatory domains to activate alternative signaling pathways. Systematic evaluation of Fas-TNFR switch receptors identified Fas-CD40 as one of the most effective configurations, markedly enhancing CAR-T cell proliferation, persistence, and cytotoxicity. This enhanced activity is attributed to the unique signaling properties of the CD40 intracellular domain, including TRAF6 recruitment and induction of a robust NF-κB-dependent activation and survival program compared to other TNFR family endodomains (145). Clinical translation of this strategy is ongoing with TK-6302, a PRAME-targeted TCR-T product that integrates a high-affinity TCR, a chimeric single-chain CD8 co-receptor, and a Fas-based checkpoint-converting switch receptor to improve T cell engraftment, fitness, and survival in solid tumors (146).

## Re−wiring the TME with ligand-ectodomain fusion proteins

The tumor microenvironment (TME) is a complex ecosystem comprising tumor cells, fibroblasts, endothelial and stromal cells, immune cells, the extracellular matrix, and a milieu of diverse cytokines and chemokines. In contrast to normal tissue, the TME features abnormal vasculature, chronic inflammation, immunosuppression, and physicochemical stresses such as hypoxia and acidosis. These conditions collectively promote tumor growth, invasion, metastasis, and resistance to therapy (147). Within this dynamic environment, immune cell populations play pivotal roles in determining tumor fate. Effector CD8^+^ and CD4^+^ T cells, NK cells, DCs, and pro-inflammatory macrophages drive tumor rejection and control, while Tregs, MDSCs, immunosuppressive macrophages, and pro-tumor neutrophils facilitate immune evasion (148). The relative abundance and functional state of these cells underpin the “hot” versus “cold” tumor paradigm, which predicts responsiveness to immunotherapy (149).

Among these immune cells, innate immune cells are particularly plastic and responsive to cues from tumor and stroma. Tumor-derived signals can program innate cells toward either tumor-promoting or tumor-fighting phenotypes, but more often these cues drive them into immunosuppressive states that blunt T cell function and reinforce a “cold” TME. Therapeutic strategies aimed at re-polarizing innate immune populations are therefore a promising approach to restore anti-tumor immunity and reshape the TME.

### CD40L-based costimulatory receptors

CD40 ligand (CD40L, CD154) is a type II transmembrane protein in the TNF superfamily, essential for activating dendritic cell and enabling efficient T cell priming. After TCR engagement, CD40L is rapidly and transiently upregulated, with mRNA peaking within 1–2 hours and surface expression reaching maximal levels by roughly 4–6 hours, before being downregulated as part of a tightly regulated activation program, most clearly defined in CD4^+^ T cells but also observed in subsets of CD8^+^ T cells (150, 151). This transient expression enables activated CD4^+^ and CD8^+^ T cells to engage CD40 on APCs. CD40 signaling in dendritic cells drives their maturation, upregulates the costimulatory molecules CD80 and CD86, enhances antigen processing, and stimulates IL-12 production. Collectively, these effects provide the signals required for robust CD8^+^ T cell priming, effector function, and memory formation. In macrophages, CD40 engagement, such as via agonistic anti-CD40 monoclonal antibodies, upregulates proinflammatory genes and promotes anti-tumor programming. In tumor-bearing mice, treatment with agonistic anti-CD40 antibodies converts macrophages to an antitumor phenotype and promotes macrophage-dependent immune responses (152, 153). Agonist anti-CD40 antibodies have shown therapeutic potential both in preclinical and clinical studies (154). However, broader clinical application has been limited by dose-dependent toxicities, suboptimal pharmacokinetics, poor tumor penetration, and difficulties achieving tumor-restricted delivery (155).

Curran et al. demonstrated that constitutive expression of CD40L on T cells can turn them from purely targeted killers into local immune modulators (156). CD40L−engineered T cells not only exhibited enhanced proliferation and increased production of Th1−type cytokines but also boosted the immunogenicity of CD40^+^ tumor cells by upregulating costimulatory, adhesion, Fas, and HLA molecules. In addition, CD40L^+^ T cells also promoted DC maturation and induced IL−12 production, “licensing” APCs and enabling a broader endogenous antitumor response. When engineered with a CD19 CAR, CD40L^+^ CAR-T cells displayed enhanced cytotoxicity against CD40^+^ tumors. *In vivo*, Curran et al. showed that CD40L/1928z CAR-T cells modestly prolonged the survival in a xenotransplant model of CD19^+^ systemic lymphoma. However, it is important to note that these studies were performed in an immunocompromised mouse strain where human CD40L does not bind to murine CD40. This limitation precludes activation of endogenous myeloid and lymphoid compartments, likely underestimating the full potential of this strategy.

**Figure 3:**
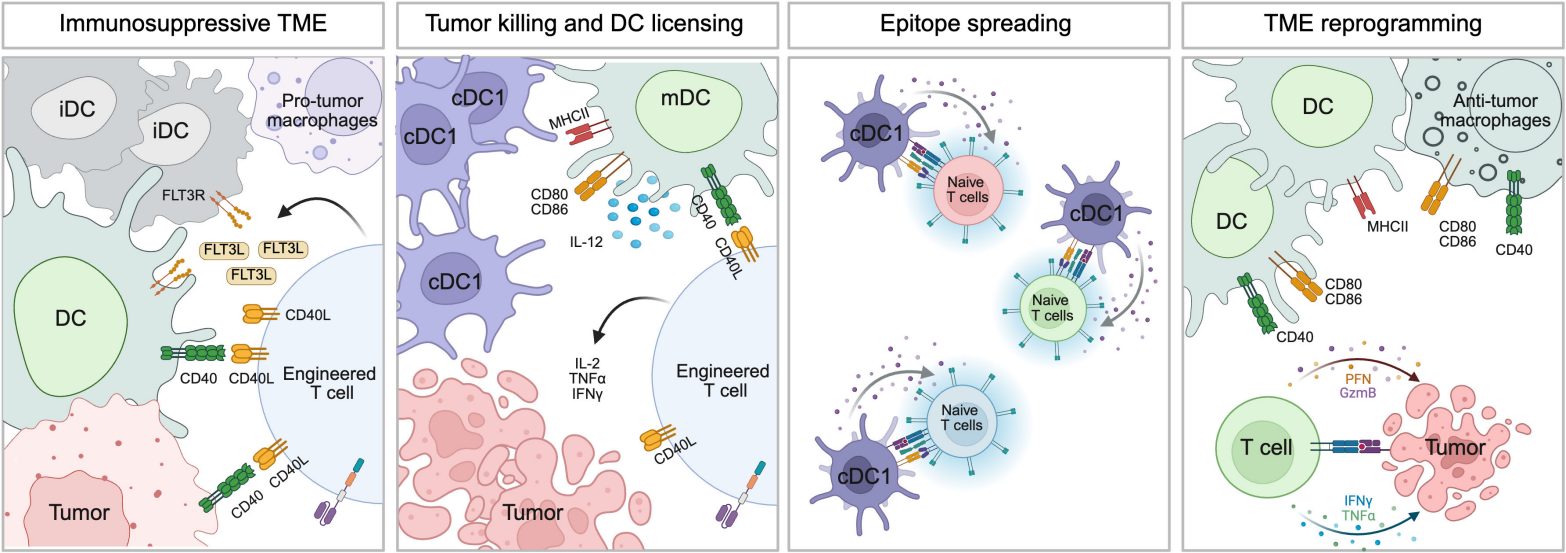
Engineering fusion proteins to reprogram the immunosuppressive TME. Immature dendritic cells (iDC) and pro-tumor macrophages support tumor growth and fail to effectively prime T cells. Engineered T cells expressing CD40L and FLT3L fusion proteins engage CD40 on dendritic cells (DC) promoting IL−12 production, upregulation of MHC class II and CD80/CD86, and enhanced tumor cell killing. Licensed cDC1 cross-present tumor antigens, driving epitope spreading and the priming of diverse naïve T cell clones. These processes remodel the TME, increasing activated DC and cDC1 populations, inducing anti-tumor macrophage polarization, and supporting cytotoxic T cell function through perforin/granzyme and inflammatory cytokine secretion.

Using an immunocompetent lymphoma model, Kuhn et al. demonstrated that CD19- CAR-T cells engineered with constitutive CD40L expression achieved superior control of CD19^+^CD40^+^ lymphoma compared to conventional CAR-T cells, despite no enhancement of tumor cell lysis *in vitro* (157). This enhanced efficacy was linked to increased DCs frequency at the tumor site, induction of DC licensing in the spleen and lymph nodes, enhanced T cell priming, and greater infiltration of both CD4^+^ and CD8^+^ tumor-infiltrating lymphocytes (TILs), along with elevated IFNγ and TNF-α production. Loss of efficacy in CD40^-^/^-^ mice confirmed that sustained CD40 engagement on host cells is required for effective immune remodeling. Additionally, the study noted that CD40 is expressed on many hematologic and solid tumors, allowing CD40L to signal directly in malignant cells. Notably, membrane−bound CD40L triggered caspase−8−dependent apoptosis in CD40^+^ carcinomas, whereas soluble CD40L provided weaker or qualitatively different signals. In follow-up work (158), the same group explored how CD40L/CD19 CAR-T cells reshape the dendritic cell landscape in the TME. They found that CD40L/CD19 CAR-T therapy expands tumor-resident CD103^+^ conventional DC cells (cDCs), specialized cross-presenting DCs that are central for coordinating CD4^+^ and CD8^+^ effector T cell trafficking and activation, thereby reinforcing a productive, cDC1-driven antitumor axis (**Figure 3**).

Olguín Contreras et al. advanced CD40L engineering by creating CD40L-CD28 chimeric costimulatory receptors, in which the CD40L ectodomain was fused to a CD28 signaling module (159). They developed two type I constructs, each inverting the soluble CD40L ectodomain and linking it via a Gly/Ser linker and either an IgG1Fc or a shorter Fil3 spacer to the CD28 transmembrane and cytoplasmic regions, as well as a type II construct that preserved the native orientation of the CD40L’s extracellular and transmembrane domains, joining them to an inverted CD28 cytoplasmic tail for a receptor similar in size and topology to endogenous CD40L. Human T cells coexpressing these CD40L-CD28 fusion proteins alongside HLA-A2-restricted TCRs (TCR-T58, TCR-D115, or TCR53) showed progressive loss of surface expression over approximately two weeks, suggesting that further optimization is necessary to stabilize these receptors. Nevertheless, even transient expression was sufficient to enhance TCR-driven cytokine secretion and antigen-specific cytotoxicity against CD40^+^ targets, demonstrating that high surface density is not required for functional signaling. Importantly, the CD40L ectodomain within these fusions retained its ability to promote maturation and activation of B cells and DCs. This highlights the potential of CD40L-CD28 switch receptors as versatile tools to boost T cell costimulation and engage antigen-presenting cells in the TME, while underscoring the need for ongoing improvements in receptor stability and a deeper understanding of costimulatory signal integration.

### FLT3L expression in engineered adoptive T cell therapies

Fms-like tyrosine kinase 3 ligand (FLT3L) is a critical hematopoietic growth factor that signals through the FLT3 receptor (CD135) to drive the proliferation, differentiation, and survival of multipotent progenitors and dendritic cell precursors (160). Neutralization of FLT3L results in a profound reduction in classical DCs, including both the cross−presenting cDC1 subset and cDC2, as well as plasmacytoid DCs (pDCs), underscoring its non−redundant role in DC ontogeny and immune homeostasis (161). This central role has motivated extensive interest in leveraging FLT3L to enhance antigen presentation, cross-priming, and endogenous T cell responses in cancer immunotherapy.

FLT3L administration as a soluble recombinant cytokine has been tested in numerous preclinical cancer models (160). Systemic administration of FLT3L robustly expands DC populations, especially the cDC1 subsets capable of cross-presenting tumor antigens to CD8^+^ T cells (162, 163). Despite these promising features, soluble FLT3L administration has several limitations: recombinant FLT3L has a short serum half-life, leading to only transient DC expansion that often requires repeated or high-dose systemic administration to sustain biologic activity (164, 165). Moreover, systemic delivery leads to broad expansion of DCs and hematopoietic progenitors across multiple compartments rather than preferential enrichment at tumor sites or tumor-draining lymph nodes, potentially diluting its impact on productive tumor antigen cross-presentation (160). These limitations have constrained the efficacy of soluble FLT3L as a standalone immunotherapy and highlight the need for strategies that localize and temporally coordinate FLT3L−driven DC expansion with innate activation and T−cell effector function.

FLT3L expression in adoptive T cell therapies was first reported by Lai et al, who engineered T cells to secrete FLT3L using TCR- and CAR-based constructs for adoptive cell transfer (166). This research showed that FLT3L-secreting T cells markedly expanded intratumoral cDC1s, especially when combined with poly(I:C) and anti-4-1BB. This expansion led to increased activation of host DCs and T cells, better tumor control, and, most notably, epitope spreading (**Figure 3**). These findings provided strong preclinical evidence that increasing DC populations via FLT3L can help counteract antigen-escape mechanisms that often limit the efficacy of targeted adoptive therapies in solid tumor models. Mechanistically, FLT3L expression affected not only the infused cells but also altered the TME by recruiting and activating host APCs to prime a broader repertoire of T cells. A more recent study by Swan et al. engineered EGFRvIII-targeted CAR-T cells to express secreted FLT3L alone or in combination with IL-7 in a syngeneic, antigen-heterogeneous glioblastoma model (167). Unlike many preclinical CAR studies that use immunodeficient hosts or uniform antigen expression, this model comprised approximately 50% EGFRvIII-positive tumor cells and did not rely on full lymphodepletion prior to CAR infusion. In this setting, IL-7 expression proved critical for enhancing intratumoral CAR-T cell abundance, consistent with well-established role of IL-7 in supporting T cell survival, expansion, and memory formation. Co-expression of FLT3L with IL-7 modestly increased the numbers of cDCs. Survival analyses revealed that CAR-T cells expressing IL-7, either alone or with FLT3L, significantly improved overall survival compared to conventional CAR-T cells, which performed poorly in this stringent model. The addition of FLT3L did not dramatically change CAR-T cell abundance but appeared to enhance DC recruitment and cross-presentation, suggesting a collaborative role with IL-7 in mobilizing both the engineered and endogenous arms of the antitumor response.

FLT3L expression in engineered T cell therapies is a strategically attractive approach to extend the benefits of adoptive transfer beyond direct cytotoxicity. However, an important clinical consideration is that the lymphodepleting regimens used to precondition patients for adoptive T cell transfer not only facilitate engraftment of the infused cells but also transiently deplete endogenous antitumor lymphocytes and other immune effectors. Reducing epitope-spreading targets such as endogenous tumor-specific T cells and their antigen-presenting counterparts may diminish the efficacy of engineering strategies such as FLT3L delivery. As such, cooperative factors such as TLR agonists, costimulatory agonists, and exogenous or secreted cytokines offer synergistic signals that help repopulate and activate depleted immune compartments. Lai et al. (166) and Swan et al. (167). demonstrated the value of these combination strategies in inducing a diverse antitumor response, balancing the need for T cell engraftment with the maintenance of robust endogenous immune responses. By fostering DC expansion and enhancing antigen presentation, FLT3L can promote epitope spreading and broader immune engagement, addressing two of the most persistent barriers in solid tumor therapy: antigen heterogeneity and immunosuppressive microenvironments.

### Overexpressing costimulatory ligands on engineered T cells

One strategy to overcome deficient costimulation in the TME is to overexpress native costimulatory ligands on the surface of therapeutic T cells. Stephan et al. implemented this strategy by engineering primary human CD8 TCR-T cells to coexpress CD80 (the ligand for CD28) and 4-1BBL (the ligand for 4-1BB), reasoning that such T cells could function as self-sufficient vehicles of costimulation even when encountering tumor cells lacking these molecules (168). CD80/4−1BBL−armed T cells showed robust proliferation upon repeated antigen stimulation and rejected large systemic tumors in immunodeficient mice. Mechanistically, the coexpressed ligands engaged CD28 and 4−1BB at the immunological synapse to enhance T cell activation and survival. The authors further demonstrated both auto−costimulation within individual T cells and trans−costimulation of bystander T cells.

Building on ligand-based concepts, Dobrin et al. introduced a novel synthetic costimulatory approach. Rather than displaying separate ligands on the cell surface, 80BB acts as both ligand and receptor, simultaneously engaging CD28 and delivering 4-1BB signaling within engineered T cells (169). This approach improved antitumor activity in HLA-independent TCR-engineered T cells, conventional TCR-T cells, and TIL. Functional studies showed that 80BB boosts CD3-dependent T cell responses, including cytokine production, proliferation, and tumor suppression across various settings. Notably, 80BB also functions as a switch receptor: when it binds the inhibitory checkpoint CTLA−4, it delivers an activating 4−1BB costimulatory signal, and loss of endogenous CTLA−4 in 80BB−expressing T cells diminishes tumor control *in vivo*, confirming that CTLA−4 ligation provides an agonistic input into 80BB activity.

The glucocorticoid-induced TNF receptor (GITR; TNFRSF18) is an inducible costimulatory receptor found on activated CD4^+^ and CD8^+^ T cells and is persistently expressed at high levels on Tregs (170). Engagement of GITR by its ligand, GITRL (TNFSF18), promotes effector T cell proliferation, cytokine production, and survival while reducing susceptibility to activation−induced cell death; at the same time, GITR signaling can attenuate Treg suppressive activity, making this pathway an attractive immunotherapy target (171, 172). To localize GITR costimulation to the TME, Tan et al. engineered PSMA-BB-z CAR-T cells to express membrane-bound GITRL, which increased proliferation, cytokine secretion, and antitumor activity relative to control T cells (173). GITRL-expressing CAR-T cells were resistant to Treg-mediated suppression, and upon engaging GITR on Tregs, impaired their suppressive function, resulting in tumors with higher effector-to-Treg ratios and decreased intratumoral Treg activity. Collectively, these studies show that overexpressing costimulatory ligands or ligand-mimetic receptors on therapeutic T cells can both augment effector function and locally relieve immune suppression, supporting ligand-based TNFR stimulation as a spatially controlled strategy to improve adoptive T cell therapy.

While GITRL−engineered CAR−T cells primarily modulate Treg activity through direct cell−to−cell contact, additional soluble factors in the TME, most notably transforming growth factor beta (TGF−β), also contribute to Treg induction and maintenance. TGF−β facilitates the differentiation of naïve CD4^+^ T cells into Foxp3^+^ regulatory T cells, suppresses the proliferation and cytotoxicity of conventional CD4^+^ and CD8^+^ T cells, impairs DCs and NK cell function, and promotes pro−tumor phenotypes in macrophages and neutrophils (174). To counteract TGF-β–mediated immunosuppression, several T cell engineering strategies have been developed. One approach involves equipping T cells with a TGFβR−derived switch receptor, in which the TGF−β receptor ectodomain is fused to a 4−1BB costimulatory domain. This design enables TGF−β engagement to deliver a costimulatory signal, thereby supporting T cell activation, proliferation, and antitumor activity in TGF−β−rich tumors. In a complementary approach, T cells can be engineered to secrete a TGF-β–blocking antibody fragment based on the Fresolimumab (GC1008) single-chain variable fragment, which binds and neutralizes extracellular TGF-β in the local microenvironment (175). Su and Thelen et al. describe an alternative strategy that employs an immunomodulatory receptor linking TGF−β receptor ectodomains directly to IL−2Rβ/γ signaling motifs. This design redirects TGF−β input into STAT5−driven survival and expansion pathways, eliminating the requirement for exogenous cytokines (176). Collectively, these approaches indicate that ligand−ectodomain fusion logic can be applied beyond contact−dependent TNFR ligands to include dominant soluble suppressive cues such as TGF−β, thereby reducing Treg−mediated suppression and improving the functional capacity of engineered T cells.

## Selection of switch and fusion receptors in adoptive T cell immunotherapy

Optimal fusion receptor design should directly address the dominant immunologic barriers in a given tumor and be tailored to the specific therapeutic platform. When immune suppression is primarily mediated by checkpoint ligands on tumor and myeloid cells, checkpoint−targeted switch receptors, such as PD−1− or TIGIT−based constructs, can convert widespread inhibitory signals into localized co−stimulation without introducing new antigen specificities. Conversely, switch receptors derived from Fas or CD200R are more appropriate when death−receptor signaling or myeloid−driven regulatory pathways constrain T cell persistence, trigger activation−induced cell death, or promote tolerogenic APC phenotypes. A biomarker−guided strategy is therefore highly desirable, although not strictly required, as PD−L1–negative tumors can still respond to PD−1 pathway blockade. Quantitative and spatial profiling of ligands, such as PD−L1, CD155, CD200, and FasL, across tumor, stromal, and immune compartments should inform the choice of ectodomain. Simultaneously, characterizing infiltrating myeloid subsets offers insight into which pathways are functionally dominant. However, ligand expression alone is insufficient; functional assessments of T cell apoptosis, exhaustion, and suppression are necessary to identify the checkpoints and death pathways that most strongly restrict antitumor immunity. When designing ligand-based fusion constructs, such as those incorporating CD40L or FLT3L, it is critical to account for tumor context. Tumor histology, baseline DC abundance, and the extent to which efficacy depends on antigen spreading should guide whether local delivery of immune-modulatory ligands is likely to meaningfully amplify endogenous antitumor responses. Finally, receptor architecture must be calibrated to the expected persistence and clinical context of the cellular product. Short−lived allogeneic platforms may tolerate more potent pro−inflammatory or pro−survival signaling modules. In contrast, long−lived autologous products generally require stricter control to prevent tonic signaling, lineage skewing, or delayed on−target toxicity.

## Conclusion

The tumor microenvironment is a complex and dynamic ecosystem. Achieving durable responses will likely require engineered T cell therapies that modulate multiple suppressive pathways and stromal interactions, rather than targeting a single axis. Thus far, most optimization of switch receptors, ligand-based fusion proteins, and costimulatory domains has been performed in CD19-directed CAR-T cell systems, underscoring the need to extend and validate these design principles across diverse CAR backbones and antigen targets that more accurately reflect the challenges posed by solid tumors.

Switch−receptor constructs and secreted or tethered immune−modulatory fusion proteins operate through fundamentally different mechanisms and are not functionally interchangeable; parameters such as extracellular domain length and orientation, transmembrane choice, and the tendency to dimerize or form higher-order assemblies can markedly influence signal quality, specificity, and *in vivo* efficacy. Furthermore, these structure–activity relationships are modulated by the TME, including ligand abundance, spatial organization, and competing inhibitory signals. Systematic evaluation of how individual design features impacts antitumor activity across different tumor settings is therefore essential.

Within costimulatory modules, a “Goldilocks” model of CAR-T cell activation has emerged in which both insufficient and excessive costimulation can lead to dysfunction, poor persistence, or toxicity instead of durable tumor control. Additionally, CD4^+^ and CD8^+^ CAR−T cells play distinct yet complementary functions. CD4^+^ CAR−T cells typically secrete higher levels of Th1−polarizing cytokines, including IFNγ, TNF-α, and IL-2, supporting overall proliferation and differentiation, while CD8^+^ CAR−T cells exhibit greater per−cell cytolytic activity against tumor targets (177). Preclinical and early clinical studies show that products with defined CD4:CD8 ratios, promote expansion, maintain CD8^+^ effector function, and achieve synergistic antitumor effects compared to CD8−only products (178). These results suggest that costimulatory strategies, including the incorporation of switch receptors, should account for potential differences between CD4^+^ and CD8^+^ CAR-T cells, recognizing that some signals (such as 4-1BB-mediated costimulation) can effectively support both subsets, whereas others may preferentially enhance one over the other. Although numerous innovative designs have demonstrated promising efficacy in preclinical settings, their clinical translation remains challenging. Bridging these gaps requires ongoing optimization of efficacy, clinically relevant head-to-head comparisons, systematic toxicity assessments, and evaluation in CAR-T products for diverse tumor antigen targets to meet key clinical needs.

Safety considerations are a major factor in advancing these engineering platforms to the clinic. Ligand-inducible ON switches and logic-gated circuits offer powerful opportunities to externally tune CAR-T activity and improve tumor selectivity (179). However, certain implementations may still exhibit leaky activity, on-target effects in normal tissues that express the gating antigen, or context-dependent and unpredictable responses to exogenous trigger molecules. Conversely, costimulatory fusions that enhance pre-existing antigen recognition can substantially boost responses to tumor cells, but in some configurations may also promote tonic signaling or increase the risk of on-target, off-tumor effects in tissues with low-level antigen expression. Innovative safety strategies include multi-antigen logic gating, incorporation of suicide or drug-controlled OFF switches, restriction of potent payloads to the immunological synapse, and rigorous antigen selection and dose-escalation protocols in early-phase studies. Switch- and fusion-based approaches complement established methods such as checkpoint or death-receptor gene knockout and cytokine armoring. While gene edits that abrogate inhibitory pathways or boost prosurvival factors can confer constitutive resistance to suppression, they generally lack the spatial and temporal precision of switch receptors, which can deliver antigen- and context-dependent signaling to reduce toxicity and better preserve normal regulatory mechanisms.

## References

[1] Brown CE Mackall CL . Car t cell therapy: inroads to response and resistance. Nat Rev Immunol. (2019) 19:73–4. doi: 10.1038/s41577-018-0119-y 30631206

[2] Zugasti I Espinosa-Aroca L Fidyt K Mulens-Arias V Diaz-Beya M Juan M . Car-t cell therapy for cancer: current challenges and future directions. Signal Transd Targ Ther. (2025) 10:210. doi: 10.1038/s41392-025-02269-w 40610404 PMC12229403

[3] Vitanza NA Ronsley R Choe M Seidel K Huang W Rawlings-Rhea SD . Intracerebroventricular b7-h3-targeting car t cells for diffuse intrinsic pontine glioma: a phase 1 trial. Nat Med. (2025) 31:861–8. doi: 10.1038/s41591-024-03451-3 39775044 PMC11922736

[4] Vitanza NA Wilson AL Huang W Seidel K Brown C Gustafson JA . Intraventricular b7-h3 car t cells for diffuse intrinsic pontine glioma: preliminary first-in-human bioactivity and safety. Cancer Discov. (2023) 13:114–31. doi: 10.1158/2159-8290.CD-22-0750 36259971 PMC9827115

[5] Del Bufalo F De Angelis B Caruana I Del Baldo G De Ioris MA Serra A . Gd2-cart01 for relapsed or refractory high-risk neuroblastoma. N Engl J Med. (2023) 388:1284–95. doi: 10.1056/NEJMoa2210859 37018492

[6] Rapoport AP Stadtmauer EA Binder-Scholl GK Goloubeva O Vogl DT Lacey SF . Ny-eso-1-specific tcr-engineered t cells mediate sustained antigen-specific antitumor effects in myeloma. Nat Med. (2015) 21:914–21. doi: 10.1038/nm.3910 26193344 PMC4529359

[7] Mullard A . Fda approves first tumour-infiltrating lymphocyte (til) therapy, bolstering hopes for cell therapies in solid cancers. Nat Rev Drug Discov. (2024) 23:238. doi: 10.1038/d41573-024-00035-1 38374249

[8] Galon J Bruni D . Approaches to treat immune hot, altered and cold tumours with combination immunotherapies. Nat Rev Drug Discov. (2019) 18:197–218. doi: 10.1038/s41573-018-0007-y 30610226

[9] Junttila MR de Sauvage FJ . Influence of tumour micro-environment heterogeneity on therapeutic response. Nature. (2013) 501:346–54. doi: 10.1038/nature12626 24048067

[10] Petrova V Annicchiarico-Petruzzelli M Melino G Amelio I . The hypoxic tumour microenvironment. Oncogenesis. (2018) 7:10. doi: 10.1038/s41389-017-0011-9 29362402 PMC5833859

[11] Kuipers MT Kersten MJ . Cd19-directed chimeric antigen receptor t-cell therapy: what can we learn from the haematologist? Lupus Sci Med. (2025) 12. doi: 10.1136/lupus-2024-001157 39832905 PMC11751780

[12] Park JH Geyer MB Brentjens RJ . Cd19-targeted car t-cell therapeutics for hematologic Malignancies: interpreting clinical outcomes to date. Blood. (2016) 127:3312–20. doi: 10.1182/blood-2016-02-629063 27207800 PMC4929923

[13] Labanieh L Mackall CL . Car immune cells: design principles, resistance and the next generation. Nature. (2023) 614:635–48. doi: 10.1038/s41586-023-05707-3 36813894

[14] Teng MW Ngiow SF von Scheidt B McLaughlin N Sparwasser T Smyth MJ . Conditional regulatory t-cell depletion releases adaptive immunity preventing carcinogenesis and suppressing established tumor growth. Cancer Res. (2010) 70:7800–9. doi: 10.1158/0008-5472.CAN-10-1681 20924111

[15] O'Brien SA Orf J Skrzypczynska KM Tan H Kim J DeVoss J . Activity of tumor-associated macrophage depletion by csf1r blockade is highly dependent on the tumor model and timing of treatment. Cancer Immunol Immunother. (2021) 70:2401–10. doi: 10.1007/s00262-021-02861-3 33511454 PMC8289806

[16] Guo J Kent A Davila E . Chimeric non-antigen receptors in t cell-based cancer therapy. J Immunother Cancer. (2021) 9. doi: 10.1136/jitc-2021-002628 34344725 PMC8336119

[17] Rane R Li F Williams A Jayadev A Tran NL Winkles JA . Chimeric switch and inverted cytokine receptors in t cell therapy: reprogramming t cells to overcome immune suppression in the solid tumor microenvironment. Front Immunol. (2025) 16:1662238. doi: 10.3389/fimmu.2025.1662238 41132663 PMC12540331

[18] Smith-Garvin JE Koretzky GA Jordan MS . T cell activation. Annu Rev Immunol. (2009) 27:591–619. doi: 10.1146/annurev.immunol.021908.132706 19132916 PMC2740335

[19] Schwartz RH . T cell anergy. Annu Rev Immunol. (2003) 21:305–34. doi: 10.1146/annurev.immunol.21.120601.141110 12471050

[20] Esensten JH Helou YA Chopra G Weiss A Bluestone JA . Cd28 costimulation: from mechanism to therapy. Immunity. (2016) 44:973–88. doi: 10.1016/j.immuni.2016.04.020 27192564 PMC4932896

[21] Boise LH Minn AJ Noel PJ June CH Accavitti MA Lindsten T . Cd28 costimulation can promote t cell survival by enhancing the expression of bcl-xl. Immunity. (1995) 3:87–98. doi: 10.1016/1074-7613(95)90161-2 7621080

[22] Curtsinger JM Mescher MF . Inflammatory cytokines as a third signal for t cell activation. Curr Opin Immunol. (2010) 22:333–40. doi: 10.1016/j.coi.2010.02.013 20363604 PMC2891062

[23] Toumi R Yuzefpolskiy Y Vegaraju A Xiao H Smith KA Sarkar S . Autocrine and paracrine il-2 signals collaborate to regulate distinct phases of cd8 t cell memory. Cell Rep. (2022) 39:110632. doi: 10.1016/j.celrep.2022.110632 35417685 PMC12948409

[24] Ward-Kavanagh LK Lin WW Sedy JR Ware CF . The tnf receptor superfamily in co-stimulating and co-inhibitory responses. Immunity. (2016) 44:1005–19. doi: 10.1016/j.immuni.2016.04.019 27192566 PMC4882112

[25] Croft M . Co-stimulatory members of the tnfr family: keys to effective t-cell immunity? Nat Rev Immunol. (2003) 3:609–20. doi: 10.1038/nri1148 12974476

[26] Oh HS Choi BK Kim YH Lee DG Hwang S Lee MJ . 4-1bb signaling enhances primary and secondary population expansion of cd8+ t cells by maximizing autocrine il-2/il-2 receptor signaling. PloS One. (2015) 10:e0126765. doi: 10.1371/journal.pone.0126765 25962156 PMC4427336

[27] Redmond WL Ruby CE Weinberg AD . The role of ox40-mediated co-stimulation in t-cell activation and survival. Crit Rev Immunol. (2009) 29:187–201. doi: 10.1615/critrevimmunol.v29.i3.10 19538134 PMC3180959

[28] Ruby CE Redmond WL Haley D Weinberg AD . Anti-ox40 stimulation *in vivo* enhances cd8+ memory t cell survival and significantly increases recall responses. Eur J Immunol. (2007) 37:157–66. doi: 10.1002/eji.200636428 17183611

[29] Munks MW Mourich DV Mittler RS Weinberg AD Hill AB . 4-1bb and ox40 stimulation enhance cd8 and cd4 t-cell responses to a dna prime, poxvirus boost vaccine. Immunology. (2004) 112:559–66. doi: 10.1111/j.1365-2567.2004.01917.x 15270726 PMC1782516

[30] Harris DT Hager MV Smith SN Cai Q Stone JD Kruger P . Comparison of t cell activities mediated by human tcrs and cars that use the same recognition domains. J Immunol. (2018) 200:1088–100. doi: 10.4049/jimmunol.1700236 29288199 PMC5780198

[31] Wesolowski J Alzogaray V Reyelt J Unger M Juarez K Urrutia M . Single domain antibodies: promising experimental and therapeutic tools in infection and immunity. Med Microbiol Immunol. (2009) 198:157–74. doi: 10.1007/s00430-009-0116-7 19529959 PMC2714450

[32] Bannas P Hambach J Koch-Nolte F . Nanobodies and nanobody-based human heavy chain antibodies as antitumor therapeutics. Front Immunol. (2017) 8:1603. doi: 10.3389/fimmu.2017.01603 29213270 PMC5702627

[33] Cappell KM Kochenderfer JN . A comparison of chimeric antigen receptors containing cd28 versus 4-1bb costimulatory domains. Nat Rev Clin Oncol. (2021) 18:715–27. doi: 10.1038/s41571-021-00530-z 34230645

[34] Cook MS King E Flaherty KR Siddika K Papa S Benjamin R . Car-t cells containing cd28 versus 4-1bb co-stimulatory domains show distinct metabolic profiles in patients. Cell Rep. (2025) 44:115973. doi: 10.1016/j.celrep.2025.115973 40650909

[35] Administration FaD . Approved cellular and gene therapy products 2025. Available online at: https://www.fda.gov/vaccines-blood-biologics/cellular-gene-therapy-products/approved-cellular-and-gene-therapy-products (Accessed February 14, 2026).

[36] Kawalekar OU O'Connor RS Fraietta JA Guo L McGettigan SE Posey AD . Distinct signaling of coreceptors regulates specific metabolism pathways and impacts memory development in car t cells. Immunity. (2016) 44:380–90. doi: 10.1016/j.immuni.2016.01.021 26885860 PMC13498396

[37] Guedan S Chen X Madar A Carpenito C McGettigan SE Frigault MJ . Icos-based chimeric antigen receptors program bipolar th17/th1 cells. Blood. (2014) 124:1070–80. doi: 10.1182/blood-2013-10-535245 24986688 PMC4133482

[38] Weinkove R George P Dasyam N McLellan AD . Selecting costimulatory domains for chimeric antigen receptors: functional and clinical considerations. Clin Transl Immunol. (2019) 8:e1049. doi: 10.1002/cti2.1049 31110702 PMC6511336

[39] Volkov DV Stepanova VM Yaroshevich IA Gabibov AG Rubtsov YP . The impact of the intracellular domains of chimeric antigenic receptors on the properties of car t-cells. Acta Naturae. (2025) 17:4–17. doi: 10.32607/actanaturae.27728 41122323 PMC12536988

[40] Hombach AA Heiders J Foppe M Chmielewski M Abken H . Ox40 costimulation by a chimeric antigen receptor abrogates cd28 and il-2 induced il-10 secretion by redirected cd4(+) t cells. Oncoimmunology. (2012) 1:458–66. doi: 10.4161/onci.19855 22754764 PMC3382912

[41] Guercio M Orlando D Di Cecca S Sinibaldi M Boffa I Caruso S . Cd28.ox40 co-stimulatory combination is associated with long *in vivo* persistence and high activity of car.cd30 t-cells. Haematologica. (2021) 106:987–99. doi: 10.3324/haematol.2019.231183 32381575 PMC8018158

[42] Kaczanowska S Murty T Alimadadi A Contreras CF Duault C Subrahmanyam PB . Immune determinants of car-t cell expansion in solid tumor patients receiving gd2 car-t cell therapy. Cancer Cell. (2024) 42:35–51:e8. doi: 10.1016/j.ccell.2023.11.011 38134936 PMC10947809

[43] Moreno-Cortes E Franco-Fuquen P Garcia-Robledo JE Forero J Booth N Castro JE . Icos and ox40 tandem co-stimulation enhances car t-cell cytotoxicity and promotes t-cell persistence phenotype. Front Oncol. (2023) 13:1200914. doi: 10.3389/fonc.2023.1200914 37719008 PMC10502212

[44] Webb GJ Hirschfield GM Lane PJ . Ox40, ox40l and autoimmunity: a comprehensive review. Clin Rev Allergy Immunol. (2016) 50:312–32. doi: 10.1007/s12016-015-8498-3 26215166

[45] Julamanee J Terakura S Umemura K Adachi Y Miyao K Okuno S . Composite cd79a/cd40 co-stimulatory endodomain enhances cd19car-t cell proliferation and survival. Mol Ther. (2021) 29:2677–90. doi: 10.1016/j.ymthe.2021.04.038 33940156 PMC8417513

[46] Prinzing B Schreiner P Bell M Fan Y Krenciute G Gottschalk S . Myd88/cd40 signaling retains car t cells in a less differentiated state. JCI Insight. (2020) 5. doi: 10.1172/jci.insight.136093 33148882 PMC7710311

[47] Mata M Gerken C Nguyen P Krenciute G Spencer DM Gottschalk S . Inducible activation of myd88 and cd40 in car t cells results in controllable and potent antitumor activity in preclinical solid tumor models. Cancer Discov. (2017) 7:1306–19. doi: 10.1158/2159-8290.CD-17-0263 28801306 PMC5780189

[48] Foster AE Mahendravada A Shinners NP Chang WC Crisostomo J Lu A . Regulated expansion and survival of chimeric antigen receptor-modified t cells using small molecule-dependent inducible myd88/cd40. Mol Ther. (2017) 25:2176–88. doi: 10.1016/j.ymthe.2017.06.014 28697888 PMC5589084

[49] Stein MN Dumbrava EE Teply BA Gergis US Guiterrez ME Reshef R . Psca-targeted bpx-601 car t cells with pharmacological activation by rimiducid in metastatic pancreatic and prostate cancer: a phase 1 dose escalation trial. Nat Commun. (2024) 15:10743. doi: 10.1038/s41467-024-53220-6 39737899 PMC11685978

[50] Tang L Pan S Wei X Xu X Wei Q . Arming car-t cells with cytokines and more: innovations in the fourth-generation car-t development. Mol Ther. (2023) 31:3146–62. doi: 10.1016/j.ymthe.2023.09.021 37803832 PMC10638038

[51] Kagoya Y Tanaka S Guo T Anczurowski M Wang CH Saso K . A novel chimeric antigen receptor containing a JAK-STAT signaling domain mediates superior antitumor effects. Nat Med. (2018) 24:352–9. doi: 10.1038/nm.4478 29400710 PMC5839992

[52] Chapuis AG Egan DN Bar M Schmitt TM McAfee MS Paulson KG . T cell receptor gene therapy targeting WT1 prevents acute myeloid leukemia relapse post-transplant. Nat Med. (2019) 25:1064–72. doi: 10.1038/s41591-019-0472-9 31235963 PMC6982533

[53] Wang T Navenot JM Rafail S Kurtis C Carroll M Van Kerckhoven M . Identifying MAGE-A4-positive tumors for TCR T cell therapies in HLA-A *02-eligible patients. Mol Ther Methods Clin Dev. (2024) 32:101265. doi: 10.1016/j.omtm.2024.101265 38872830 PMC11170170

[54] Krakow EF Brault M Summers C Cunningham TM Biernacki MA Black RG . HA-1-targeted T-cell receptor T-cell therapy for recurrent leukemia after hematopoietic stem cell transplantation. Blood. (2024) 144:1069–82. doi: 10.1182/blood.2024024105 38683966 PMC11406181

[55] Chandran SS Klebanoff CA . T cell receptor-based cancer immunotherapy: Emerging efficacy and pathways of resistance. Immunol Rev. (2019) 290:127–47. doi: 10.1111/imr.12772 31355495 PMC7027847

[56] Baulu E Gardet C Chuvin N Depil S . TCR-engineered T cell therapy in solid tumors: State of the art and perspectives. Sci Adv. (2023) 9:eadf3700. doi: 10.1126/sciadv.adf3700 36791198 PMC9931212

[57] Zhang S Tang TH Kinsella S Mazziotta F Schweizer MT McAfee MS . A CD8alphabeta co-receptor modified to contain an intracellular CD28 signaling tail enhances TCR-engineered T cell function independent of solid-tumor-associated co-stimulatory ligands. Nat Commun. (2026) 17:753. doi: 10.1038/s41467-025-67446-5 41484084 PMC12820041

[58] Boucher JC Li G Kotani H Cabral ML Morrissey D Lee SB . CD28 costimulatory domain-targeted mutations enhance chimeric antigen receptor T-cell function. Cancer Immunol Res. (2021) 9:62–74. doi: 10.1158/2326-6066.CIR-20-0253 33188139 PMC7864379

[59] Prosser ME Brown CE Shami AF Forman SJ Jensen MC . Tumor PD-L1 co-stimulates primary human CD8(+) cytotoxic T cells modified to express a PD1:CD28 chimeric receptor. Mol Immunol. (2012) 51:263–72. doi: 10.1016/j.molimm.2012.03.023 22503210

[60] Oda SK Daman AW Garcia NM Wagener F Schmitt TM Tan X . A CD200R-CD28 fusion protein appropriates an inhibitory signal to enhance T-cell function and therapy of murine leukemia. Blood. (2017) 130:2410–9. doi: 10.1182/blood-2017-04-777052 29042364 PMC5709784

[61] Oda SK Anderson KG Ravikumar P Bonson P Garcia NM Jenkins CM . A Fas-4-1BB fusion protein converts a death to a pro-survival signal and enhances T cell therapy. J Exp Med. (2020) 217. doi: 10.1084/jem.20191166 32860705 PMC7953733

[62] Hui E Cheung J Zhu J Su X Taylor MJ Wallweber HA . T cell costimulatory receptor CD28 is a primary target for PD-1-mediated inhibition. Science. (2017) 355:1428–33. doi: 10.1126/science.aaf1292 28280247 PMC6286077

[63] Agata Y Kawasaki A Nishimura H Ishida Y Tsubata T Yagita H . Expression of the PD-1 antigen on the surface of stimulated mouse T and B lymphocytes. Int Immunol. (1996) 8:765–72. doi: 10.1093/intimm/8.5.765 8671665

[64] Sharpe AH Pauken KE . The diverse functions of the PD1 inhibitory pathway. Nat Rev Immunol. (2018) 18:153–67. doi: 10.1038/nri.2017.108 28990585

[65] Liu R Zeng LW Li HF Shi JG Zhong B Shu HB . PD-1 signaling negatively regulates the common cytokine receptor gamma chain via MARCH5-mediated ubiquitination and degradation to suppress anti-tumor immunity. Cell Res. (2023) 33:923–39. doi: 10.1038/s41422-023-00890-4 37932447 PMC10709454

[66] Freeman GJ Long AJ Iwai Y Bourque K Chernova T Nishimura H . Engagement of the PD-1 immunoinhibitory receptor by a novel B7 family member leads to negative regulation of lymphocyte activation. J Exp Med. (2000) 192:1027–34. doi: 10.1084/jem.192.7.1027 11015443 PMC2193311

[67] Wherry EJ Kurachi M . Molecular and cellular insights into T cell exhaustion. Nat Rev Immunol. (2015) 15:486–99. doi: 10.1038/nri3862 26205583 PMC4889009

[68] Zou W Wolchok JD Chen L . PD-L1 (B7-H1) and PD-1 pathway blockade for cancer therapy: Mechanisms, response biomarkers, and combinations. Sci Transl Med. (2016) 8:328rv4. doi: 10.1126/scitranslmed.aad7118 26936508 PMC4859220

[69] Garon EB Rizvi NA Hui R Leighl N Balmanoukian AS Eder JP . Pembrolizumab for the treatment of non-small-cell lung cancer. N Engl J Med. (2015) 372:2018–28. doi: 10.1056/NEJMoa1501824 25891174

[70] Larkin J Chiarion-Sileni V Gonzalez R Grob JJ Rutkowski P Lao CD . Five-year survival with combined nivolumab and ipilimumab in advanced melanoma. N Engl J Med. (2019) 381:1535–46. doi: 10.1056/NEJMoa1910836 31562797

[71] Sul J Blumenthal GM Jiang X He K Keegan P Pazdur R . FDA approval summary: Pembrolizumab for the treatment of patients with metastatic non-small cell lung cancer whose tumors express programmed death-ligand 1. Oncologist. (2016) 21:643–50. doi: 10.1634/theoncologist.2015-0498 27026676 PMC4861368

[72] Sepesi B Cuentas EP Canales JR Behrens C Correa AM Vaporciyan A . Programmed death cell ligand 1 (PD-L1) is associated with survival in stage I non-small cell lung cancer. Semin Thorac Cardiovasc Surg. (2017) 29:408–15. doi: 10.1053/j.semtcvs.2017.05.008 29195578

[73] Li W Wu F Zhao S Shi P Wang S Cui D . Correlation between PD-1/PD-L1 expression and polarization in tumor-associated macrophages: A key player in tumor immunotherapy. Cytokine Growth Fact Rev. (2022) 67:49–57. doi: 10.1016/j.cytogfr.2022.07.004 35871139

[74] Lee J Ahn E Kissick HT Ahmed R . Reinvigorating exhausted T cells by blockade of the PD-1 pathway. For Immunopathol Dis Therap. (2015) 6:7–17. doi: 10.1615/ForumImmunDisTher.2015014188 28286692 PMC5341794

[75] Martinez FO Gordon S . The M1 and M2 paradigm of macrophage activation: Time for reassessment. F1000Prime Rep. (2014) 6:13. doi: 10.12703/P6-13 24669294 PMC3944738

[76] Biswas SK Gangi L Paul S Schioppa T Saccani A Sironi M . A distinct and unique transcriptional program expressed by tumor-associated macrophages (defective NF-kappaB and enhanced IRF-3/STAT1 activation). Blood. (2006) 107:2112–22. doi: 10.1182/blood-2005-01-0428 16269622

[77] Karimova AF Khalitova AR Suezov R Markov N Mukhamedshina Y Rizvanov AA . Immunometabolism of tumor-associated macrophages: A therapeutic perspective. Eur J Cancer. (2025) 220:115332. doi: 10.1016/j.ejca.2025.115332 40048925

[78] Liu Y Zugazagoitia J Ahmed FS Henick BS Gettinger SN Herbst RS . Immune cell PD-L1 colocalizes with macrophages and is associated with outcome in PD-1 pathway blockade therapy. Clin Cancer Res. (2020) 26:970–7. doi: 10.1158/1078-0432.CCR-19-1040 31615933 PMC7024671

[79] Arlauckas SP Garris CS Kohler RH Kitaoka M Cuccarese MF Yang KS . *In vivo* imaging reveals a tumor-associated macrophage-mediated resistance pathway in anti-PD-1 therapy. Sci Transl Med. (2017) 9. doi: 10.1126/scitranslmed.aal3604 28490665 PMC5734617

[80] Li Z Duan D Li L Peng D Ming Y Ni R . Tumor-associated macrophages in anti-PD-1/PD-L1 immunotherapy for hepatocellular carcinoma: Recent research progress. Front Pharmacol. (2024) 15:1382256. doi: 10.3389/fphar.2024.1382256 38957393 PMC11217528

[81] Liu X Ranganathan R Jiang S Fang C Sun J Kim S . A chimeric switch-receptor targeting PD1 augments the efficacy of second-generation CAR T cells in advanced solid tumors. Cancer Res. (2016) 76:1578–90. doi: 10.1158/0008-5472.CAN-15-2524 26979791 PMC4800826

[82] Qin L Cui Y Yuan T Chen D Zhao R Li S . Co-expression of a PD-L1-specific chimeric switch receptor augments the efficacy and persistence of CAR T cells via the CD70-CD27 axis. Nat Commun. (2022) 13:6051. doi: 10.1038/s41467-022-33793-w 36229619 PMC9561169

[83] Schlenker R Olguin-Contreras LF Leisegang M Schnappinger J Disovic A Ruhland S . Chimeric PD-1:28 receptor upgrades low-avidity T cells and restores effector function of tumor-infiltrating lymphocytes for adoptive cell therapy. Cancer Res. (2017) 77:3577–90. doi: 10.1158/0008-5472.CAN-16-1922 28533272

[84] Guo JX Wu CX Wang PF Li ZJ Han S Jin W . Bioactivity and safety of chimeric switch receptor T cells in glioblastoma patients. Front Biosci (Landm Ed). (2019) 24:1158–66. doi: 10.2741/4772 31136972

[85] Supimon K Sangsuwannukul T Sujjitjoon J Chieochansin T Junking M Yenchitsomanus PT . Cytotoxic activity of anti-mucin 1 chimeric antigen receptor T cells expressing PD-1-CD28 switch receptor against cholangiocarcinoma cells. Cytotherapy. (2023) 25:148–61. doi: 10.1016/j.jcyt.2022.10.006 36396553

[86] Liu H Lei W Zhang C Yang C Wei J Guo Q . CD19-specific CAR T cells that express a PD-1/CD28 chimeric switch-receptor are effective in patients with PD-L1-positive B-cell lymphoma. Clin Cancer Res. (2021) 27:473–84. doi: 10.1158/1078-0432.CCR-20-1457 33028589

[87] Chen C Gu YM Zhang F Zhang ZC Zhang YT He YD . Construction of PD1/CD28 chimeric-switch receptor enhances anti-tumor ability of c-Met CAR-T in gastric cancer. Oncoimmunology. (2021) 10:1901434. doi: 10.1080/2162402X.2021.1901434 33854821 PMC8018404

[88] Sailer N Fetzer I Salvermoser M Braun M Brechtefeld D Krendl C . T-cells expressing a highly potent PRAME-specific T-cell receptor in combination with a chimeric PD1-41BB co-stimulatory receptor show a favorable preclinical safety profile and strong anti-tumor reactivity. Cancers (Basel). (2022) 14. doi: 10.3390/cancers14081998 35454906 PMC9030144

[89] Ma Q He X Zhang B Guo F Ou X Yang Q . A PD-L1-targeting chimeric switch receptor enhances efficacy of CAR-T cell for pleural and peritoneal metastasis. Signal Transd Targ Ther. (2022) 7:380. doi: 10.1038/s41392-022-01198-2 36402752 PMC9675732

[90] Carturan A Angelos MG Guruprasad P Patel RP Pajarillo R Lee AE . Harnessing the CD2 axis to broaden and enhance the efficacy of CAR T-cell therapies. Blood. (2026) 147(16):1842–56. doi: 10.1182/blood.2025031409 41490267 PMC13181175

[91] Verhagen J Sabatos CA Wraith DC . The role of CTLA-4 in immune regulation. Immunol Lett. (2008) 115:73–4. doi: 10.1016/j.imlet.2007.10.010 18035425 PMC2629540

[92] Berg M Zavazava N . Regulation of CD28 expression on CD8+ T cells by CTLA-4. J Leukoc Biol. (2008) 83:853–63. doi: 10.1189/jlb.0107065 18162511

[93] Hossen MM Ma Y Yin Z Xia Y Du J Huang JY . Current understanding of CTLA-4: From mechanism to autoimmune diseases. Front Immunol. (2023) 14:1198365. doi: 10.3389/fimmu.2023.1198365 37497212 PMC10367421

[94] Yin L Schneider H Rudd CE . Short cytoplasmic SDYMNM segment of CD28 is sufficient to convert CTLA-4 to a positive signaling receptor. J Leukoc Biol. (2003) 73:178–82. doi: 10.1189/jlb.0702365 12525576

[95] Shin JH Park HB Oh YM Lim DP Lee JE Seo HH . Positive conversion of negative signaling of CTLA4 potentiates antitumor efficacy of adoptive T-cell therapy in murine tumor models. Blood. (2012) 119:5678–87. doi: 10.1182/blood-2011-09-380519 22538857

[96] Park HB Lee JE Oh YM Lee SJ Eom HS Choi K . CTLA4-CD28 chimera gene modification of T cells enhances the therapeutic efficacy of donor lymphocyte infusion for hematological Malignancy. Exp Mol Med. (2017) 49:e360. doi: 10.1038/emm.2017.104 28751785 PMC5565951

[97] Lin S Cheng L Ye W Li S Zheng D Qin L . Chimeric CTLA4-CD28-CD3z T cells potentiate antitumor activity against CD80/CD86-positive B cell Malignancies. Front Immunol. (2021) 12:642528. doi: 10.3389/fimmu.2021.642528 33868277 PMC8050336

[98] Prinz LF Riet T Neureuther DF Lennartz S Chrobok D Hubbe H . An anti-CD19/CTLA-4 switch improves efficacy and selectivity of CAR T cells targeting CD80/86-upregulated DLBCL. Cell Rep Med. (2024) 5:101421. doi: 10.1016/j.xcrm.2024.101421 38340727 PMC10897622

[99] Clayton KL Haaland MS Douglas-Vail MB Mujib S Chew GM Ndhlovu LC . T cell Ig and mucin domain-containing protein 3 is recruited to the immune synapse, disrupts stable synapse formation, and associates with receptor phosphatases. J Immunol. (2014) 192:782–91. doi: 10.4049/jimmunol.1302663 24337741 PMC4214929

[100] Rangachari M Zhu C Sakuishi K Xiao S Karman J Chen A . Bat3 promotes T cell responses and autoimmunity by repressing Tim-3-mediated cell death and exhaustion. Nat Med. (2012) 18:1394–400. doi: 10.1038/nm.2871 22863785 PMC3491118

[101] Zhang J Wang L Guo H Kong S Li W He Q . The role of Tim-3 blockade in the tumor immune microenvironment beyond T cells. Pharmacol Res. (2024) 209:107458. doi: 10.1016/j.phrs.2024.107458 39396768

[102] Wolf Y Anderson AC Kuchroo VK . TIM3 comes of age as an inhibitory receptor. Nat Rev Immunol. (2020) 20:173–85. doi: 10.1038/s41577-019-0224-6 31676858 PMC7327798

[103] Zhao S Wang C Lu P Lou Y Liu H Wang T . Switch receptor T3/28 improves long-term persistence and antitumor efficacy of CAR-T cells. J Immunother Cancer. (2021) 9. doi: 10.1136/jitc-2021-003176 34853180 PMC8638458

[104] Wang C Wang C Liu H Zhao S Qiu J Li P . Immune function assessing of TIM3/CD28-modified CD19 CAR-T cells and general CD19 CAR-T cells through a high-throughput single-cell microarray platform. Interdiscip Med. (2024) 2:e20230030. doi: 10.1002/INMD.20230030 41531421

[105] Blaeschke F Ortner E Stenger D Mahdawi J Apfelbeck A Habjan N . Design and evaluation of TIM-3-CD28 checkpoint fusion proteins to improve anti-CD19 CAR T-cell function. Front Immunol. (2022) 13:845499. doi: 10.3389/fimmu.2022.845499 35464394 PMC9018974

[106] Yu X Harden K Gonzalez LC Francesco M Chiang E Irving B . The surface protein TIGIT suppresses T cell activation by promoting the generation of mature immunoregulatory dendritic cells. Nat Immunol. (2009) 10:48–57. doi: 10.1038/ni.1674 19011627

[107] Stanietsky N Rovis TL Glasner A Seidel E Tsukerman P Yamin R . Mouse TIGIT inhibits NK-cell cytotoxicity upon interaction with PVR. Eur J Immunol. (2013) 43:2138–50. doi: 10.1002/eji.201243072 23677581 PMC3863769

[108] Ge Z Peppelenbosch MP Sprengers D Kwekkeboom J . TIGIT, the next step towards successful combination immune checkpoint therapy in cancer. Front Immunol. (2021) 12:699895. doi: 10.3389/fimmu.2021.699895 34367161 PMC8339559

[109] Hoogi S Eisenberg V Mayer S Shamul A Barliya T Cohen CJ . A TIGIT-based chimeric co-stimulatory switch receptor improves T-cell anti-tumor function. J Immunother Cancer. (2019) 7:243. doi: 10.1186/s40425-019-0721-y 31500665 PMC6734436

[110] Blessin NC Simon R Kluth M Fischer K Hube-Magg C Li W . Patterns of TIGIT expression in lymphatic tissue, inflammation, and cancer. Dis Markers. (2019) 2019:5160565. doi: 10.1155/2019/5160565 30733837 PMC6348838

[111] Cui H Hamad M Elkord E . TIGIT in cancer: from mechanism of action to promising immunotherapeutic strategies. Cell Death Dis. (2025) 16:664. doi: 10.1038/s41419-025-07984-4 40890162 PMC12402281

[112] Zhao J Dong J Deng C Zhang Q Sun S Li H . Enhancing T cell anti-tumor efficacy with a PD1-TIGIT chimeric immune-checkpoint switch receptor. Oncoimmunology. (2023) 12:2265703. doi: 10.1080/2162402X.2023.2265703 37808405 PMC10557556

[113] Gorczynski RM . CD200 and its receptors as targets for immunoregulation. Curr Opin Investig Drugs. (2005) 6:483–8 15912961

[114] Wright GJ Cherwinski H Foster-Cuevas M Brooke G Puklavec MJ Bigler M . Characterization of the CD200 receptor family in mice and humans and their interactions with CD200. J Immunol. (2003) 171:3034–46. doi: 10.4049/jimmunol.171.6.3034 12960329

[115] Tonks A Hills R White P Rosie B Mills KI Burnett AK . CD200 as a prognostic factor in acute myeloid leukaemia. Leukemia. (2007) 21:566–8. doi: 10.1038/sj.leu.2404559 17252007

[116] Xin C Zhu J Gu S Yin M Ma J Pan C . CD200 is overexpressed in neuroblastoma and regulates tumor immune microenvironment. Cancer Immunol Immunother. (2020) 69:2333–43. doi: 10.1007/s00262-020-02589-6 32514618 PMC11027654

[117] Petermann KB Rozenberg GI Zedek D Groben P McKinnon K Buehler C . CD200 is induced by ERK and is a potential therapeutic target in melanoma. J Clin Invest. (2007) 117:3922–9. doi: 10.1172/JCI32163 18008004 PMC2075477

[118] Love JE Thompson K Kilgore MR Westerhoff M Murphy CE Papanicolau-Sengos A . CD200 expression in neuroendocrine neoplasms. Am J Clin Pathol. (2017) 148:236–42. doi: 10.1093/ajcp/aqx071 28821198 PMC5848429

[119] Zhang S Cherwinski H Sedgwick JD Phillips JH . Molecular mechanisms of CD200 inhibition of mast cell activation. J Immunol. (2004) 173:6786–93. doi: 10.4049/jimmunol.173.11.6786 15557172

[120] Mihrshahi R Barclay AN Brown MH . Essential roles for Dok2 and RasGAP in CD200 receptor-mediated regulation of human myeloid cells. J Immunol. (2009) 183:4879–86. doi: 10.4049/jimmunol.0901531 19786546 PMC2788151

[121] Liu JQ Hu A Zhu J Yu J Talebian F Bai XF . CD200-CD200R pathway in the regulation of tumor immune microenvironment and immunotherapy. Adv Exp Med Biol. (2020) 1223:155–65. doi: 10.1007/978-3-030-35582-1_8 32030689 PMC7339106

[122] Coles SJ Hills RK Wang EC Burnett AK Man S Darley RL . Expression of CD200 on AML blasts directly suppresses memory T-cell function. Leukemia. (2012) 26:2148–51. doi: 10.1038/leu.2012.77 22430635 PMC3460216

[123] Minas K Liversidge J . Is the CD200/CD200 receptor interaction more than just a myeloid cell inhibitory signal? Crit Rev Immunol. (2006) 26:213–30. doi: 10.1615/critrevimmunol.v26.i3.20 16928187 PMC2446434

[124] Schulze-Osthoff K Ferrari D Los M Wesselborg S Peter ME . Apoptosis signaling by death receptors. Eur J Biochem. (1998) 254:439–59. doi: 10.1046/j.1432-1327.1998.2540439.x 9688254

[125] Suda T Takahashi T Golstein P Nagata S . Molecular cloning and expression of the Fas ligand, a novel member of the tumor necrosis factor family. Cell. (1993) 75:1169–78. doi: 10.1016/0092-8674(93)90326-l 7505205

[126] Schneider P Holler N Bodmer JL Hahne M Frei K Fontana A . Conversion of membrane-bound Fas(CD95) ligand to its soluble form is associated with downregulation of its proapoptotic activity and loss of liver toxicity. J Exp Med. (1998) 187:1205–13. doi: 10.1084/jem.187.8.1205 9547332 PMC2212219

[127] Suda T Hashimoto H Tanaka M Ochi T Nagata S . Membrane Fas ligand kills human peripheral blood T lymphocytes, and soluble Fas ligand blocks the killing. J Exp Med. (1997) 186:2045–50. doi: 10.1084/jem.186.12.2045 9396774 PMC2199173

[128] Roberts AI Devadas S Zhang X Zhang L Keegan A Greeneltch K . The role of activation-induced cell death in the differentiation of T-helper-cell subsets. Immunol Res. (2003) 28:285–93. doi: 10.1385/IR:28:3:285 14713720

[129] Alderson MR Tough TW Davis-Smith T Braddy S Falk B Schooley KA . Fas ligand mediates activation-induced cell death in human T lymphocytes. J Exp Med. (1995) 181:71–7. doi: 10.1084/jem.181.1.71 7528780 PMC2191813

[130] Ju ST Panka DJ Cui H Ettinger R el-Khatib M Sherr DH . Fas(CD95)/FasL interactions required for programmed cell death after T-cell activation. Nature. (1995) 373:444–8. doi: 10.1038/373444a0 7530337

[131] Griffith TS Brunner T Fletcher SM Green DR Ferguson TA . Fas ligand-induced apoptosis as a mechanism of immune privilege. Science. (1995) 270:1189–92. doi: 10.1126/science.270.5239.1189 7502042

[132] Reimer T Herrnring C Koczan D Richter D Gerber B Kabelitz D . FasL : Fas ratio--a prognostic factor in breast carcinomas. Cancer Res. (2000) 60:822–8 10706087

[133] Munakata S Enomoto T Tsujimoto M Otsuki Y Miwa H Kanno H . Expressions of Fas ligand and other apoptosis-related genes and their prognostic significance in epithelial ovarian neoplasms. Br J Cancer. (2000) 82:1446–52. doi: 10.1054/bjoc.1999.1073 10780525 PMC2363379

[134] Ito Y Monden M Takeda T Eguchi H Umeshita K Nagano H . The status of Fas and Fas ligand expression can predict recurrence of hepatocellular carcinoma. Br J Cancer. (2000) 82:1211–7. doi: 10.1054/bjoc.1999.1065 10735508 PMC2363358

[135] Neuber K Eidam B . Expression of Fas ligand (CD95L) in primary Malignant melanoma and melanoma metastases is associated with overall survival. Onkologie. (2006) 29:361–5. doi: 10.1159/000094355 16974112

[136] Lin Y Liu L Zhang T Liu J . Functional investigation of Fas ligand expressions in human non-small cell lung cancer cells and its clinical implications. Ann Thorac Surg. (2013) 95:412–8. doi: 10.1016/j.athoracsur.2012.08.012 23021303

[137] Modiano JF Bellgrau D . Fas ligand based immunotherapy: A potent and effective neoadjuvant with checkpoint inhibitor properties, or a systemically toxic promoter of tumor growth? Discov Med. (2016) 21:109–16 27011046

[138] Wada A Tada Y Kawamura K Takiguchi Y Tatsumi K Kuriyama T . The effects of FasL on inflammation and tumor survival are dependent on its expression levels. Cancer Gene Ther. (2007) 14:262–7. doi: 10.1038/sj.cgt.7701008 17053813

[139] Peter ME . DICE: A novel tumor surveillance mechanism-a new therapy for cancer? Cell Cycle. (2014) 13:1373–8. doi: 10.4161/cc.28673 24690893 PMC4050134

[140] Hadji A Ceppi P Murmann AE Brockway S Pattanayak A Bhinder B . Death induced by CD95 or CD95 ligand elimination. Cell Rep. (2014) 7:208–22. doi: 10.1016/j.celrep.2014.02.035 24656822 PMC4083055

[141] Kunkele A Johnson AJ Rolczynski LS Chang CA Hoglund V Kelly-Spratt KS . Functional tuning of CARs reveals signaling threshold above which CD8+ CTL antitumor potency is attenuated due to cell Fas-FasL-dependent AICD. Cancer Immunol Res. (2015) 3:368–79. doi: 10.1158/2326-6066.CIR-14-0200 25576337

[142] Yamamoto TN Lee PH Vodnala SK Gurusamy D Kishton RJ Yu Z . T cells genetically engineered to overcome death signaling enhance adoptive cancer immunotherapy. J Clin Invest. (2019) 129:1551–65. doi: 10.1172/JCI121491 30694219 PMC6436880

[143] Stromnes IM Schmitt TM Hulbert A Brockenbrough JS Nguyen H Cuevas C . T cells engineered against a native antigen can surmount immunologic and physical barriers to treat pancreatic ductal adenocarcinoma. Cancer Cell. (2015) 28:638–52. doi: 10.1016/j.ccell.2015.09.022 26525103 PMC4724422

[144] Anderson KG Oda SK Bates BM Burnett MG Rodgers Suarez M Ruskin SL . Engineering adoptive T cell therapy to co-opt Fas ligand-mediated death signaling in ovarian cancer enhances therapeutic efficacy. J Immunother Cancer. (2022) 10. doi: 10.1136/jitc-2021-003959 35264436 PMC8915280

[145] McKenzie C El-Kholy M Parekh F Robson M Lamb K Allen C . Novel Fas-TNFR chimeras that prevent Fas ligand-mediated kill and signal synergistically to enhance CAR T cell efficacy. Mol Ther Nucleic Acids. (2023) 32:603–21. doi: 10.1016/j.omtn.2023.04.017 37200859 PMC10185706

[146] Triller G Salei N Stettnisch L Selck C Heichenko M Srinivasan V . 329 TK-6302, a supercharged PRAME TCR-T cell therapy containing a high affinity TCR, a costimulatory CD8 coreceptor and a FAS-based switch receptor, demonstrates preclinical safety and efficacy. J Immunother Cancer. (2025) 13. doi: 10.1136/jitc-2025-SITC2025.0329

[147] de Visser KE Joyce JA . The evolving tumor microenvironment: From cancer initiation to metastatic outgrowth. Cancer Cell. (2023) 41:374–403. doi: 10.1016/j.ccell.2023.02.016 36917948

[148] Lv B Wang Y Ma D Cheng W Liu J Yong T . Immunotherapy: Reshape the tumor immune microenvironment. Front Immunol. (2022) 13:844142. doi: 10.3389/fimmu.2022.844142 35874717 PMC9299092

[149] Wu B Zhang B Li B Wu H Jiang M . Cold and hot tumors: from molecular mechanisms to targeted therapy. Signal Transd Targ Ther. (2024) 9:274. doi: 10.1038/s41392-024-01979-x 39420203 PMC11491057

[150] Koguchi Y Thauland TJ Slifka MK Parker DC . Preformed CD40 ligand exists in secretory lysosomes in effector and memory CD4+ T cells and is quickly expressed on the cell surface in an antigen-specific manner. Blood. (2007) 110:2520–7. doi: 10.1182/blood-2007-03-081299 17595332 PMC1988919

[151] Tay NQ Lee DCP Chua YL Prabhu N Gascoigne NRJ Kemeny DM . CD40L expression allows CD8(+) T cells to promote their own expansion and differentiation through dendritic cells. Front Immunol. (2017) 8:1484. doi: 10.3389/fimmu.2017.01484 29163545 PMC5672143

[152] Hoves S Ooi CH Wolter C Sade H Bissinger S Schmittnaegel M . Rapid activation of tumor-associated macrophages boosts preexisting tumor immunity. J Exp Med. (2018) 215:859–76. doi: 10.1084/jem.20171440 29436396 PMC5839760

[153] Beatty GL Chiorean EG Fishman MP Saboury B Teitelbaum UR Sun W . CD40 agonists alter tumor stroma and show efficacy against pancreatic carcinoma in mice and humans. Science. (2011) 331:1612–6. doi: 10.1126/science.1198443 21436454 PMC3406187

[154] Mantovani A Allavena P Marchesi F Garlanda C . Macrophages as tools and targets in cancer therapy. Nat Rev Drug Discov. (2022) 21:799–820. doi: 10.1038/s41573-022-00520-5 35974096 PMC9380983

[155] Chames P Van Regenmortel M Weiss E Baty D . Therapeutic antibodies: successes, limitations and hopes for the future. Br J Pharmacol. (2009) 157:220–33. doi: 10.1111/j.1476-5381.2009.00190.x 19459844 PMC2697811

[156] Curran KJ Seinstra BA Nikhamin Y Yeh R Usachenko Y van Leeuwen DG . Enhancing antitumor efficacy of chimeric antigen receptor T cells through constitutive CD40L expression. Mol Ther. (2015) 23:769–78. doi: 10.1038/mt.2015.4 25582824 PMC4395796

[157] Kuhn NF Purdon TJ van Leeuwen DG Lopez AV Curran KJ Daniyan AF . CD40 ligand-modified chimeric antigen receptor T cells enhance antitumor function by eliciting an endogenous antitumor response. Cancer Cell. (2019) 35:473–88:e6. doi: 10.1016/j.ccell.2019.02.006 30889381 PMC6428219

[158] Kuhn NF Lopez AV Li X Cai W Daniyan AF Brentjens RJ . CD103(+) cDC1 and endogenous CD8(+) T cells are necessary for improved CD40L-overexpressing CAR T cell antitumor function. Nat Commun. (2020) 11:6171. doi: 10.1038/s41467-020-19833-3 33268774 PMC7710757

[159] Olguin-Contreras LF Mendler AN Popowicz G Hu B Noessner E . Double strike approach for tumor attack: Engineering T cells using a CD40L:CD28 chimeric co-stimulatory switch protein for enhanced tumor targeting in adoptive cell therapy. Front Immunol. (2021) 12:750478. doi: 10.3389/fimmu.2021.750478 34912334 PMC8666660

[160] Cueto FJ Sancho D . The Flt3L/Flt3 axis in dendritic cell biology and cancer immunotherapy. Cancers (Basel). (2021) 13. doi: 10.3390/cancers13071525 33810248 PMC8037622

[161] Bartczak A Sims D Zerrouki K Naiman B Qadri A Hansen AM . FLT3L neutralization reduces dendritic cell numbers, T cell activation, and salivary gland lymphocyte infiltration in the NOD.H2h4 Sjogren's mouse model. J Immunol. (2025) 214:2557–63. doi: 10.1093/jimmun/vkaf220 40902038

[162] Svensson-Arvelund J Cuadrado-Castano S Pantsulaia G Kim K Aleynick M Hammerich L . Expanding cross-presenting dendritic cells enhances oncolytic virotherapy and is critical for long-term anti-tumor immunity. Nat Commun. (2022) 13:7149. doi: 10.1038/s41467-022-34791-8 36418317 PMC9684150

[163] Theisen D Murphy K . The role of cDC1s *in vivo*: CD8 T cell priming through cross-presentation. F1000Res. (2017) 6:98. doi: 10.12688/f1000research.9997.1 28184299 PMC5288679

[164] Anandasabapathy N Breton G Hurley A Caskey M Trumpfheller C Sarma P . Efficacy and safety of CDX-301, recombinant human Flt3L, at expanding dendritic cells and hematopoietic stem cells in healthy human volunteers. Bone Marrow Transplant. (2015) 50:924–30. doi: 10.1038/bmt.2015.74 25915810 PMC4532305

[165] Bhardwaj N Friedlander PA Pavlick AC Ernstoff MS Gastman BR Hanks BA . Flt3 ligand augments immune responses to anti-DEC-205-NY-ESO-1 vaccine through expansion of dendritic cell subsets. Nat Cancer. (2020) 1:1204–17. doi: 10.1038/s43018-020-00143-y 35121932

[166] Lai J Mardiana S House IG Sek K Henderson MA Giuffrida L . Adoptive cellular therapy with T cells expressing the dendritic cell growth factor Flt3L drives epitope spreading and antitumor immunity. Nat Immunol. (2020) 21:914–26. doi: 10.1038/s41590-020-0676-7 32424363

[167] Swan SL Mehta N Ilich E Shen SH Wilkinson DS Anderson AR . IL7 and IL7 Flt3L co-expressing CAR T cells improve therapeutic efficacy in mouse EGFRvIII heterogeneous glioblastoma. Front Immunol. (2023) 14:1085547. doi: 10.3389/fimmu.2023.1085547 36817432 PMC9936235

[168] Stephan MT Ponomarev V Brentjens RJ Chang AH Dobrenkov KV Heller G . T cell-encoded CD80 and 4-1BBL induce auto- and transcostimulation, resulting in potent tumor rejection. Nat Med. (2007) 13:1440–9. doi: 10.1038/nm1676 18026115

[169] Dobrin A Lindenbergh PL Shi Y Perica K Xie H Jain N . Synthetic dual co-stimulation increases the potency of HIT and TCR-targeted cell therapies. Nat Cancer. (2024) 5:760–73. doi: 10.1038/s43018-024-00744-x 38503896 PMC11921049

[170] Nocentini G Ronchetti S Cuzzocrea S Riccardi C . GITR/GITRL: more than an effector T cell co-stimulatory system. Eur J Immunol. (2007) 37:1165–9. doi: 10.1002/eji.200636933 17407102

[171] Pascutti MF Geerman S Slot E van Gisbergen KP Boon L Arens R . Enhanced CD8 T cell responses through GITR-mediated costimulation resolve chronic viral infection. PloS Pathog. (2015) 11:e1004675. doi: 10.1371/journal.ppat.1004675 25738498 PMC4349659

[172] Knee DA Hewes B Brogdon JL . Rationale for anti-GITR cancer immunotherapy. Eur J Cancer. (2016) 67:1–10. doi: 10.1016/j.ejca.2016.06.028 27591414

[173] Tan B Tu C Xiong H Xu Y Shi X Zhang X . GITRL enhances cytotoxicity and persistence of CAR-T cells in cancer therapy. Mol Ther. (2025) 33:2789–800. doi: 10.1016/j.ymthe.2025.01.036 39863927 PMC12172191

[174] Liu S Ren J Ten Dijke P . Targeting TGFbeta signal transduction for cancer therapy. Signal Transd Targ Ther. (2021) 6:8. doi: 10.1038/s41392-020-00436-9 33414388 PMC7791126

[175] Matikhina T Cohen CJ . Targeting TGFbeta with chimeric switch receptor and secreted trap to improve T cells anti-tumor activity. Front Immunol. (2024) 15:1460266. doi: 10.3389/fimmu.2024.1460266 39512355 PMC11540659

[176] Su Y Thelen A Wirth LV Jenkins CM Mak SR Chen DG . A TGF-betaR/IL-2R immunomodulatory fusion protein transforms immunosuppression into T cell activation to enhance adoptive T cell therapy. Proc Natl Acad Sci USA. (2025) 122:e2516951122. doi: 10.1073/pnas.2516951122 40986340 PMC12501114

[177] Cadinanos-Garai A Flugel CL Cheung A Jiang E Vaissie A Abou-El-Enein M . High-dimensional temporal mapping of CAR T cells reveals phenotypic and functional remodeling during manufacturing. Mol Ther. (2025) 33:2291–309. doi: 10.1016/j.ymthe.2025.04.006 40315840 PMC12126796

[178] Sommermeyer D Hudecek M Kosasih PL Gogishvili T Maloney DG Turtle CJ . Chimeric antigen receptor-modified T cells derived from defined CD8+ and CD4+ subsets confer superior antitumor reactivity *in vivo*. Leukemia. (2016) 30:492–500. doi: 10.1038/leu.2015.247 26369987 PMC4746098

[179] Roybal KT Rupp LJ Morsut L Walker WJ McNally KA Park JS . Precision tumor recognition by T cells with combinatorial antigen-sensing circuits. Cell. (2016) 164:770–9. doi: 10.1016/j.cell.2016.01.011 26830879 PMC4752902

